# Migration or ethnic minority status and risk of autism spectrum disorders and intellectual disability: systematic review

**DOI:** 10.1093/eurpub/ckaa108

**Published:** 2020-10-13

**Authors:** Maki Morinaga, Dheeraj Rai, Anna-Clara Hollander, Nuhamin Petros, Christina Dalman, Cecilia Magnusson

**Affiliations:** 1 Department of Global Public Health, Karolinska Institutet, Stockholm, Sweden; 2 National Institute for Health Research Biomedical Research Centre, University of Bristol, Bristol, UK; 3 Centre for Academic Mental Health, Population Health Sciences, Bristol Medical School, University of Bristol, Bristol, UK; 4 Avon and Wiltshire Partnership National Health Service Mental Health Trust, Bristol, UK; 5 National Swedish Prevention of Mental Ill-Health and Suicide (NASP), Karolinska Institutet, Stockholm, Sweden

## Abstract

**Background:**

There is an emerging evidence that the migration and the ethnic minority status are associated with the risks of autism spectrum disorder (ASD) and intellectual disability (ID). This systematic review aimed to investigate whether associations are specific to ASD or ID; whether and which migration-related or ethnically determined factors are associated with the risk of ASD and ID; and what mechanisms may explain these risks.

**Methods:**

A systematic literature search was conducted using Embase, Medline and PsycINFO for studies reporting on the risks of ASD and/or ID among migrants, descendants of migrants and/or ethnic minorities. Risks of any ASD, ASD + ID, ASD – ID and any ID were reviewed in relation to migration and ethnic minority status, with consideration to the study quality. In addition, possible underlying mechanisms suggested in the included studies were summarized.

**Results:**

Thirty-five studies were included. The summarized evidence indicated an increased risk of ASD + ID and a decreased risk of ASD – ID in migrants, descendants of migrants and ethnic minorities. These associations appeared more pronounced among children of migrant mothers, with origin in low-income countries, and among descendants of migrants. Data on ID were scarce. Suggested mechanisms explaining the increased risks of ASD + ID included environmental factors acting *in utero* and genetic factors (including consanguinity), while ascertainment bias was proposed to account for the lowered risks of diagnosed ASD – ID.

**Conclusion:**

Migration-related factors acting *in utero* and/or associated with origin in low-income countries may be important in the ASD + ID aetiology, although further confirmative studies are needed.

## Introduction

Autism spectrum disorders (ASD) and intellectual disability (ID) are developmental conditions with an early onset and long-term difficulties in various domains. People with ASD typically have persistent deficits in social communication and interaction, as well as restricted and repetitive patterns of behaviour, interests or activities.^1^ Those with ID have global cognitive/intellectual impairments along with difficulties in adaptive functioning.^1^ Co-occurrence of ASD and ID is common. Over a third (32–43%) of individuals with ASD are diagnosed with ID,^2^^,^^3^ and the prevalence of ASD among people with ID ranges from 17% to 30%.^4^^,^^5^ The aetiology of ASD and ID is complex and only partly understood.^6^ Although the heritability of ASD and ID is considerable,^7^ environmental factors and gene–environment interactions are also recognized as having an impact in their aetiology.^8^^,^^9^

A migrant is an individual who migrated and has resided in a foreign country for more than one year^10^ and an ethnic minority is a group within a country or community which has different national or cultural traditions from the larger, dominant population.^11^ These two groups are broadly overlapping as most migrants become ethnic minorities in a host country (Supplementary figure S1). In addition, descendants of migrants (sometimes called second generation migrants), who are persons born in the country with at least one parent born abroad,^12^ usually maintain links with people from their parental country of origin or with the parental country and sometimes identify themselves as ethnic minority (Supplementary figure S1). Despite the differences between the groups, these terms have been used interchangeably in studies.^13^ Migrants, ethnic minorities and descendants of migrants are expanding as the number of migrants has increased substantially in the last decade, reaching 272 million worldwide in 2019.^14^ Migration is a complex and heterogenous process, involving various migration-related factors in pre-migration (e.g. region of origin and reason for migration), migration (e.g. age at migration) and post-migration stages (e.g. acculturation and socioeconomic status).^15^ Many migration-related factors are associated with poor psychological and physical health in migrants and descendants of migrants.^15^

There is an emerging evidence that the migration and the ethnic minority status are associated with the risks of ASD and ID. Several studies have shown that migrants, descendants of migrants or ethnic minorities have an increased risk of ASD with ID, but a decreased risk of ASD without ID.^2^^,^^16–20^ However, many questions remain unanswered. First, it is not known whether the observed increases in risks are specifically linked to ASD with ID or reflect a general association with ID. In addition, it has not been thoroughly explored whether severity of ASD and ID affects the association. Secondly, it is not known whether and which migration-related or ethnically determined factors are associated with the risk of ASD and ID. Especially, parental migration-related factors that children are exposed *in utero* are worth to be examined. Arguably, disentangling the role of migration from that of ethnicity is difficult. This issue is, however, important to solve since such information may unravel modifiable causes of ASD and ID. Thirdly and consequently, underlying mechanisms of any causal associations are unknown. The few existing reviews on this topic have not addressed these knowledge gaps.^13^^,^^21^^,^^22^

The aims of this study were (i) to investigate whether any association with migration or ethnic minority status is specific to ASD or ID (and/or to the severity of ASD or ID); (ii) to examine whether and which migration-related or ethnically determined factors are associated with the risk of ASD and ID; and (iii) to summarize hypotheses of what mechanisms may explain these risks. Ultimately, our goal was to elaborate implications from the body of existing evidence regarding possible causes of ASD and ID and identify specific knowledge gaps needing further investigation.

## Methods

We conducted a systematic review summarizing previous evidence of the association between migration or ethnic minority status and ASD and ID and hypotheses for underlying mechanisms. This review was guided by the Preferred Reporting Items for Systematic Reviews and Meta-Analyses (PRISMA) Statement^23^ and registered on the PROSPERO systematic review register (ID: CRD42018092589).

### Search strategy

A literature search was conducted using the electronic databases, Embase, Medline and PsycINFO, on 3 October 2018. A broad search strategy was derived that combined keywords and MeSH or Emtree terms, covering terms to characterize the outcome, e.g. terms related to ASD and ID, and terms related to exposure, e.g. migration, ethnicity and refugee (for full search strategy see Supplementary table S1). The title and abstracts of each article were screened to identify relevant articles. In addition, references cited in the articles and previous reviews were hand-searched to identify other relevant studies.

### Study inclusion and exclusion criteria

Studies were included using the following criteria: observational study of original data on prevalence or relative risks of ASD and/or ID among migrants, descendants of migrants and/or ethnic minorities; peer-reviewed human journal full-text article with abstract and written in English. Exclusion criteria were non-original articles such as reviews, and studies on ID of known cause (i.e. in individuals with genetic syndromes, such as Down syndrome or Fragile X syndrome). When multiple studies reported the same or overlapping data from the same source, only the most comprehensive report was included. All studies with different sources of data were included even if they were from the same countries with overlapping study periods.

### Data extraction

Qualitative and quantitative data were extracted regarding study year, location, study design, source of data, study population, sample size, case ascertainment, exposure (i.e. migration or ethnic minority status and place of origin) and measures of associations (i.e. prevalence or relative risks of ASD and/or ID; and their confidence intervals, *P* values and adjustment factors). In addition, authors’ hypothesized underlying mechanisms of the association between migration or ethnic minority status and ASD and ID and findings that would support or dispute the hypotheses were extracted.

### Quality assessment of the included articles

The quality of studies that met our inclusion criteria were assessed using the Newcastle Ottawa Scales for case–control and cohort studies. Studies were rated for selection of the study participants (range 0–4), comparability of cohorts/cases and controls (range 0–2) and quality of exposure/outcome ascertainment (range 0–3). The overall quality score of an article was expressed as the sum of these scores (range 0–9), with higher scores indicating greater methodological quality. We considered a score of ≥7 as a high-quality study according to common practice. The quality assessment was carried out by two reviewers (M.M. and N.P.), and any disagreement was resolved by discussion. Risk of bias across studies could not be evaluated using funnel plots, because of the heterogeneity in outcome assessments.

### Data synthesis

A meta-analysis was not conducted because of the heterogeneity in exposure and outcome assessments. Instead, included studies were systematically narratively assessed, while accounting for strengths and limitations of the individual studies.

#### Synthesis of evidence for association of migration status with ASD and/or ID

Discrepancies in risks for the various outcomes in migrants, descendants of migrants and ethnic minorities were assessed in correspondence with the first aim, to investigate whether any association with migration or ethnic minority status is specific to ASD or ID. Studies were reviewed for two categories of exposures: ‘migration’ (migrants and descendants of migrants) and ‘ethnic minority status’ (ethnic minorities) and four separate outcomes: ‘any ASD’ (all ASD regardless of co-occurring ID); ‘ASD – ID’ (Asperger syndrome and ASD without ID); ‘ASD + ID’ (infantile autism, autistic disorder and ASD with ID) and ‘any ID’ (all ID regardless of co-occurring ASD). Where relevant, any one study could be included in the review for more than one exposure and outcome. The details of the definitions for exposures and outcomes used in the included studies are available in the Supplementary tables S2 and S3.

#### Synthesis of evidence for role of migration-related or ethnically determined factors

Four strategies were used in order to address the second aim, to examine whether and which migration-related or ethnically determined factors are associated with the risk of ASD and ID. First, we examined whether risks differed within ethnic minority groups in relation to migration history. If the risks are increased in an ethnic minority group with a migration history compared with the corresponding ethnic minority group without a migration history, it may indicate that migration-related factors are more important because these two groups share ethnically determined factors. Secondly, we examined associations of the outcomes with maternal and paternal migration status in separate. Ethnically determined genetic factors are supposed to be kept virtually constant between the group with a migrant mother and the group with a migrant father, therefore, a difference in risks may depend on environmental factors acting *in utero*. Thirdly, we examined whether the risks were associated with any specific region of origin. Country or region of origin is sometimes used as a proxy for ethnicity and is also strongly associated with pre-migration factors. A strong association with origin in a specific region could possibly indicate which factors are more important in the aetiology of ASD and ID. Lastly, we compared risks among migrants and descendants of migrants, based on the notion that ethnically determined genetic factors remain constant over time while environmental factors change.

#### Synthesis of reported mechanisms

The hypotheses for underlying mechanisms of any association between migration or ethnic minority status and ASD and ID reported in the papers were categorized by outcomes and summarized depending on the results of studies.

## Results

**Figure 1:**
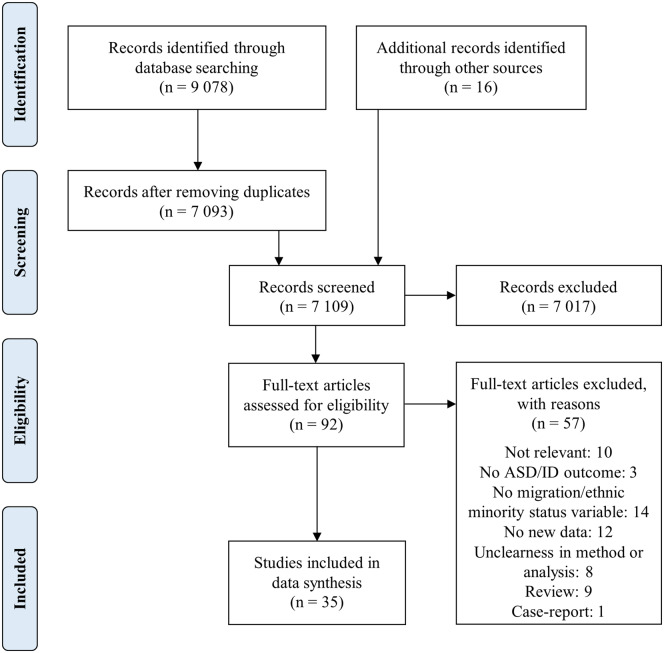
Flowchart of search and study selection *Note*: The flowchart template was adopted from the PRISMA statement (23)

Electronic searches resulted in 9078 articles. After duplicates were removed, 7093 articles were screened based on the titles and abstracts. Of these, 76 studies were retrieved for the full-text review. In addition, references cited in the articles were cross-checked, and 16 further studies were identified. Of these 92 studies, 10 were not relevant, 3 did not assess ASD or ID, 14 did not assess migration or ethnic minority status, 12 did not report new data, 8 had unclear methodology, 9 were reviews and 1 study was a case report. Hence, 35 unique studies were included in the review (figure 1).

### Study characteristics

Table 1 summarizes the main characteristics of 35 included studies. All studies were from high-income countries, including 18 (51%) conducted in Europe, 12 (34%) in the USA, 3 (9%) in Israel and 2 (6%) in Australia. Most studies (54%) were cross-sectional, while 23% and 20% were, respectively, of cohort and case–control designs. The median quality (Newcastle Ottawa Scales) score was 4 and 12 studies were of high-quality (Newcastle Ottawa Scales ≥7). In all, migration was reported from 20 studies, ethnic minority status from 18 studies and 4 studies were concerned with a combination of migration and ethnic minority status. Fifteen studies examined any ASD as the outcome, 7 studies ASD – ID, 17 studies ASD + ID and 8 studies any ID (in which only 1 study had ID without ASD as the outcome).^24^ The details of the study characteristics and results are available in Supplementary tables S4–S7.

**Table 1:** Main characteristics of the 35 studies included in the review

First author (year)	Country	Study design	Study quality with Newcastle-Ottawa Scale				Exposure			Outcome			
			Selection (max 4)	Comparability (max 2)	Exposure/outcome (max 3)	Total score (0–9)	Migration	Ethnic minority	Combination of both	Any ASD	ASD – ID	ASD + ID	Any ID
Barnevik-Olsson (2008)^36^	Sweden	Cross-sectional	★★★		★	4	○					○	
Barnevik-Olsson (2010)^37^	Sweden	Cross-sectional	★★★		★	4	○					○	
Becerra (2014)^34^	USA	Retrospective cohort	★★★	★★	★★	7	○		○			○	
Braun (2015)^33^	USA	Cross-sectional	★★★		★	4		○			○	○	○
Croen (2001)^24^	USA	Retrospective cohort	★★★★	★★	★★★	9	○	○					○
Croen (2002)^26^	USA	Retrospective cohort	★★★★	★	★★★	8	○	○		○			
Davidovitch (2013)^47^	Israel	Retrospective cohort	★★★	★	★★★	7		○		○			
Drews (1995)^31^	USA	Case–control	★	★★	★★★	6		○					○
Durkin (2017)^44^	USA	Cross-sectional	★★		★	3		○		○			
Emerson (1997)^48^	UK	Cross-sectional	★★		★	3		○					○
Emerson (2012)^32^	UK	Cross-sectional	★★★		★	4		○		○			○
Fairthorne (2017)^27^	Australia	Retrospective cohort	★★★★	★	★★	7	○	○	○		○	○	
Fernell (1998)^49^	Sweden	Cross-sectional	★★		★	3	○						○
Gillberg (1987)^40^	Sweden	Cross-sectional	★★★		★	4	○					○	
Gillberg (1996)^41^	Sweden	Cross-sectional	★★★		★	4	○					○	
Haglund (2011)^16^	Sweden	Nested case–control	★★★	★	★★★	7	○				○	○	
Hewitt (2016)^17^	USA	Cross-sectional	★★★		★	4		○		○	○	○	
Hultman (2002)^29^	Sweden	Nested case–control	★★★	★★	★★★	8	○					○	
Kamer (2004)^39^	Israel	Cross-sectional	★★		★	3	○			○			
Keen (2010)^25^	UK	Retrospective case note analysis	★★★		★	4	○	○	○	○			
Kogan (2008)^75^	USA	Cross-sectional	★★	★★		4		○		○			
Lauritsen (2005)^30^	Denmark	Cohort	★★★★	★★	★★★	9	○					○	
Lehti (2013)^18^	Finland	Nested case–control	★★★★	★★	★★★	9	○					○	
Lehti (2015)^28^	Finland	Nested case–control	★★★	★★	★★★	8	○				○		
Magnusson (2012)^19^	Sweden	Nested case–control	★★★★	★★	★★	8	○				○	○	
Maimburg (2006)^76^	Denmark	Nested case–control	★★★	★★	★★★	8	○					○	
McGrother (2002)^50^	UK	Cross-sectional	★★★		★	4		○					○
Mehta (2013)^45^	USA	Cross-sectional	★★★			3		○		○			
Morton (2002)^38^	UK	Cross-sectional	★★★		★	4		○				○	○
Pedersen (2012)^42^	USA	Cross-sectional	★★★	★★	★	6		○		○		○	
Raz (2015)^46^	Israel	Retrospective cohort	★★★★		★	5		○		○			
Singh (2013)^35^	USA	Cross-sectional	★★	★★		4			○	○			
Van der Ven (2013)^20^	Nether-lands	Retrospective cohort	★★★★	★	★★★	8	○			○	○	○	
Williams (2008)^43^	Australia	Case–control	★★	★	★★	5	○					○	
Wing (1980)^77^	UK	Cross-sectional	★★		★	3	○			○			
Yeargin-Allsopp (2003)^78^	USA	Cross-sectional	★★★	★	★	5		○		○			

★ was allocated when a feature of quality was present, ○ was allocated when a variable of exposure or outcome was present. ASD, autism spectrum disorder; ID, intellectual disability; ASD − ID, ASD without ID/Asperger’s syndrome; ASD + ID, ASD with ID/infantile autism/autistic disorder.

### Risks of ASD and ID

#### Any ASD

The results for any ASD were inconsistent between the included studies. For example, some studies, including a study that largely included cases of ‘severe’ ASD,^25^ showed an increased risk of ASD in descendants of migrants. However, the few high-quality studies on any ASD showed a decreased risk with parental migration status.^20^^,^^26^

#### ASD – ID

All seven included publications demonstrated a decreased risk of an ASD – ID diagnosis in some subgroups or entire group of migrants, descendants of migrants or ethnic minorities, although some of these studies also showed an unchanged risk in some subgroups. No study showed an increased risk in any group. All five studies on migration were of high-quality, which all showed a decreased risk of ASD – ID in migrants and descendants of migrants.^16^^,^^19^^,^^20^^,^^27^^,^^28^ There were no high-quality studies on ethnic minorities.

#### ASD + ID

Nearly all of the 17 included studies showed an increased risk of ASD + ID in some subgroups of migrants, descendants of migrants or ethnic minorities, although some of these studies also showed a decreased or unchanged risk in some groups, e.g. a decreased risk in indigenous people (i.e. Aboriginal or Torres Strait Islander) in Australia^27^ and Hispanic in the USA.^17^ The entire group of migrants and descendants of migrants were associated with an increased risk of ASD + ID according to five high-quality studies.^16^^,^^18^^,^^20^^,^^29^^,^^30^

#### Any ID

The results for any ID were inconsistent between studies when not considering the severity of ID. Three studies on ID of varying severity, including a high-quality study for ID without ASD, showed generally a decreased risk of mild to moderate ID and an unchanged or increased risk of moderate to profound ID in descendants of migrants and ethnic minorities.^24^^,^^31^^,^^32^ An exception was African-American children in the USA, shown to have an increased risk of ID regardless of the severity in three studies.^24^^,^^31^^,^^33^

### Impact of migration-related or ethnically determined factors

#### Ethnic groups with and without a migration history

There were two studies on any ASD (one from the USA and one from the UK) and one study on ASD + ID (from the USA) that compared risks within ethnic groups in relation to migration history (table 2). There was no such study on ASD – ID and any ID. The results showed that there were differences in risks of any ASD or ASD + ID between migrants and non-migrants within the same ethnic group, that, however, varied with ethnicity. All three studies showed that African American/Afro-Caribbean children with a migration history had an increased risk of any ASD or ASD + ID compared with non-migrant African-American/Afro-Caribbean children.^25^^,^^34^^,^^35^ In contrast, Hispanic children with a migration history had a decreased risk of any ASD or ASD + ID compared with non-migrant Hispanic children according to two USA studies.^34^^,^^35^ The results regarding children of Asian descent varied between studies, with increased risks of ASD + ID in migrants noted in a US high-quality study^34^ and of any ASD in a UK study^25^ and a decreased risk of any ASD in another USA study.^35^ All studies showed that risks of any ASD or ASD + ID were similar in migrant and non-migrant white children.^25^^,^^34^^,^^35^

**Table 2:** Ethnic groups with and without a migration history and risk of ASD and ID

First author (year, country)	Design (source of data, population, sample size)	Case ascertainment	Ethnic minority and migration variable	Results^b^				Adjustment factor
Becerra (2014, USA)	Retrospective cohort (Regional centres, linked to the birth register, children born 1995–2006 (*n* = 1 626 354), 7540 cases with autistic disorder, 806 of which with comorbid mental retardation, diagnosed at age 3–5 during 1998–2009)	Autistic disorder DSM-IV (ICD-9-CM code: 299.00)	Maternal race/ethnicity and whether mother is US- or foreign born	White US-born Foreign-born Black US-born Foreign-born Hispanic US-born Foreign-born Asian US-born Foreign-born	*RR (95% CI)* 1.00 1.06 (0.95–1.18) **0.68 (0.62–0.75)** **1.46 (1.18–1.80)** **0.70 (0.65–0.75)** **0.58 (0.55–0.62)** 1.02 (0.85–1.21) 1.03 (0.94–1.11)	*aRR^1^ (95% CI)* 1.00 1.02 (0.91–1.14) 1.00 (0.90–1.10) **1.59 (1.28–1.96)** 1.08 (1.00–1.17) **0.85 (0.79–0.91)** 1.03 (0.86–1.22) 0.98 (0.90–1.06)	*aRR^2^ (95% CI)* 1.00 1.05 (0.94–1.17) 1.04 (0.94–1.15) **1.65 (1.33–2.05)** **1.15 (1.06–1.24)** 1.05 (0.97–1.14) 1.02 (0.85–1.21) 1.04 (0.95–1.13)	^1^Maternal age, type of birth, parity, infant gender, year of birth, gestational age, birth weight, trimester start of prenatal care, and any pregnancy complication
		Autistic disorder DSM-IV (ICD-9-CM code: 299.00) and Mental retardation^a^ DSM-IV (ICD-9-CM codes: 317, mild; 318.0, moderate; 318.1, severe; 318.2, profound; 319, MR unspecified)		White US-born Foreign-born Black US-born Foreign-born Hispanic US-born Foreign-born Asian US-born Foreign-born	1.00 1.07 (0.73–1.56) 1.23 (0.92–1.63) **2.49 (1.41–4.42)** 0.99 (0.78–1.25) **0.76 (0.62–0.95)** 1.25 (0.72–2.18) **1.38 (1.06–1.80)**	1.00 1.06 (0.73–1.55) **1.42 (1.06–1.90)** **2.56 (1.44–4.53)** **1.30 (1.02–1.66)** 0.91 (0.73–1.14) 1.31 (0.75–2.27) **1.36 (1.04–1.77)**	1.00 1.08 (0.74–1.57) **1.47 (1.09–1.97)** **2.67 (1.50–4.74)** **1.35 (1.04–1.73)** 1.01 (0.79–1.30) 1.30 (0.75–2.27) **1.41 (1.08–1.84)**	^2^Additionally adjusted for maternal education and insurance type
Singh (2013, USA)	Cross-sectional (survey, children aged 0–17 (*n* = 91 532),? cases aged 3–17)	Questions: ‘Were you ever told by a doctor or other health-care provider that [CHILD] had autism, Asperger’s disorder, pervasive developmental disorder or other autism spectrum disorder?’ ‘Does [CHILD] currently have autism or ASD?’	Children’s migration status (migrant = foreign-born children with both migrant parents or US-born children with one or both migrant parents. Native-born = non-migrant or US-born parents) and race/ethnicity	Non-Hispanic white Native-born Migrant Non-Hispanic black Native-born Migrant Hispanic Native-born Migrant Asian Native-born Migrant Other Native-born Migrant		*Prevalence: P* < 0.05 1.26% 1.07% 0.60% 0.69% 1.40% 0.71% 0.85% 0.28% – –	*aOR (95% CI):* 1.00 0.87 (0.48–1.56) **0.43 (0.23–0.82)** 0.55 (0.18–1.74) 1.08 (0.54–2.15) 0.50 (0.21–1.18) 0.65 (0.19–2.25) **0.22 (0.10–0.51)** 1.22 (0.57–2.59) 0.57 (0.27–1.22)	Logistic regression for child’s age, sex, household composition, metropolitan/non-metropolitan residence, household poverty and education level
Keen (2010, UK)	Retrospective case note analysis (clinical service, compared with population from the census, 137 cases in Lambeth and 258 cases in Wandsworth diagnosed between 1 September 1999 to 31 August 2005)	Autism-spectrum disorders ICD-10 (2/3 had childhood autism according to ICD-10)	Mother’s ethnicity and whether mother is born in the UK or not	White UK-born Migrant Black UK-born Migrant Asian UK-born Migrant		*Adjusted rate ratio (95% CI):* Lambeth: 1.00 1.26 (0.55–2.87) **2.07 (1.14–3.75)** **8.21 (4.94–13.63)** 1.01 (0.23–4.33) **5.52 (2.57–11.83)**	*Adjusted rate ratio (95% CI):* Wandsworth: 1.00 0.73 (0.49–1.08) 0.74 (0.46–1.20) **3.86 (2.84–5.26)** –( – ) 1.13 (0.67–1.90)	Family size

a The term, mental retardation, was changed to intellectual disability in the DSM-5. b Results are presented in boldface to indicate the 95 % confidence interval does not include 1.0. ASD, autism spectrum disorder; ID, intellectual disability; MR, mental retardation; DSM, diagnostic and statistical manual of mental disorders; ICD, international classification of diseases; (a)RR, (adjusted) risk ratio; (a)OR, (adjusted) odds ratio; CI, confidence interval.

#### Maternal and paternal migration status

There were three studies on ASD + ID and one study on ASD – ID that compared risks associated with maternal and paternal migration status. There were no such studies for any ASD and any ID. Regarding ASD + ID, three high-quality studies from Nordic countries showed that maternal, but not paternal, migration was associated with an increased risk (table 3). On the other hand, there was no association between maternal or paternal migration and ASD – ID, according to a high-quality study from Finland.^28^ However, this study showed a decreased risk in children born to two migrant parents (adjusted OR = 0.2, 95% CI 0.1–0.4).^28^

**Table 3:** Maternal and paternal migration and risk of ASD + ID

First author (year)	Location	Design (source of data, population, sample size)	Case ascertainment	Results^a^	
Lehti (2013)	Finland	Nested case–control (Register, Children born 1987–2005, 1132 cases diagnosed by 2007, 4 controls/case matched by date of birth (±30 days), region of birth, sex and residence in Finland)	Childhood autism (ICD-9: code 299.0 and ICD-10: F84.0)	*OR (95% CI):* Both parents Finnish 1.0 Father only migrated 1.2 (0.8–2.0) Mother only migrated **1.8 (1.2–2.7)** Both parents migrated **1.8 (1.2–2.6)**	*Adjusted OR (95% CI):* Both parents Finnish 1.0 Father only migrated 1.3 (0.8–2.1) Mother only migrated **1.8 (1.2–2.7)** Both parents migrated **1.8 (1.2–2.7)** *Adjusted for parental age*
Haglund (2011)	Malmoe, Sweden	Nested case–control (clinical record, linked to the birth register, children born 1980–2005 (*N* = 68 964), 157 cases, controls matched by birth place and year of birth)	Autistic disorder DSM-IV or childhood autism ICD-10	*OR (95% CI):* Mother born in Sweden Father Swedish 1.0 Father not Swedish 0.8 (0.2–2.4) Mother not born in Sweden Father Swedish **2.5 (1.7–3.6)** Father not Swedish **2.9 (1.9–4.3)**	*Adjusted OR (95% CI):* Mother born in Sweden Father Swedish 1.0 Father not Swedish 0.7 (0.2–2.2) Mother not born in Sweden Father Swedish **2.1 (1.4–3.1)** Father not Swedish **2.6 (1.7–3.9)** *Adjusted for maternal age ≥40 years, birth <37 weeks and gestational age-adjusted weight Standard Deviation scores*
Lauritsen (2005)	Denmark	Cohort (register, Children born 1984–1998 (*n* = 943 664), 818 cases diagnosed by 2002)	Childhood autism (ICD-10: F84.0) or Atypical autism (ICD-10: F84.1)	*Adjusted RR (95% CI):* Both parents born in Denmark 1.00 Only father born abroad 1.15 (0.84–1.58) Only mother born abroad **1.77 (1.22–2.34)** Both parents born abroad 1.17 (0.90–1.51) *Adjusted for age and its interaction with gender, calendar year of diagnosis, maternal and paternal age, maternal, paternal and sibling psychiatric disorder, fathers identity known, urbanization and if mother and father were born in the same country*	

^a^Results are presented in boldface to indicate the 95 % confidence interval does not include 1.0. ASD, autism spectrum disorder; ID, intellectual disability; DSM, diagnostic and statistical manual of mental disorders; ICD, international classification of diseases; OR, odds ratio; RR, risk ratio; CI, confidence interval.

#### Region of origin

Overall, associations were generally more pronounced with origins from low-income countries. Asian, African and Latin American/Caribbean origins were associated with both decreased and increased risks of any ASD.^20^^,^^25^^,^^26^^,^^32^ The studies on ASD – ID showed that origins from countries such as Africa, Southern Asia and Latin America/Caribbean were associated with the decreased risk.^17^^,^^19^^,^^20^^,^^28^ The studies on ASD + ID showed that origins in Asia, Sub-Sahara Africa, Latin America/Caribbean and Former Soviet Union were associated with the increased risk.^16–19^^,^^34^^,^^36^^,^^37^ Regarding any ID, origins from most regions were associated with the decreased risk of mild-to-moderate ID,^24^^,^^32^ while Pakistani descent was associated with the increased risk of severe or profound ID.^32^^,^^38^

#### Migrants vs. descendants of migrants

The included studies researched either descendants of migrants only, or both migrants and descendants of migrants. Yet, only two studies compared risks of ASD between migrants and descendants of migrants. One study showed that none of Ethiopian-born children living in Israel (i.e. migrants) but 8.3/10 000 children born in Israel of Ethiopian extraction (i.e. descendants of migrants) was diagnosed with any ASD.^39^ Another high-quality study examined the role of timing of maternal migration in relation to birth of the index child. The study showed that the risk of ASD + ID peaked in descendants of migrants when maternal migration occurred around pregnancy (OR = 2.3, 95% CI 1.7–3.0), while the risk was not increased in migrants.^19^

### Hypotheses for underlying mechanisms

#### Increased risk of any ASD and ASD + ID

Environmental factors operating *in utero* and genetic factors were most often suggested as underlying mechanisms of the increased risk of any ASD and ASD + ID in migrants, descendants of migrants or ethnic minorities. In all, 16 studies mentioned environmental factors in pregnancy,^16^^,^^18–20^^,^^25^^,^^27–30^^,^^34^^,^^36^^,^^39^^–^^43^ including intrauterine exposure to maternal stress, infection, poor nutrition, vitamin D deficiency and toxic environmental exposures. Eight studies discussed genetic factors, including genetic variation according to race/ethnicity and consanguinity.^16^^,^^19^^,^^25^^,^^34^^,^^36^^,^^38^^,^^40^^,^^42^ Other suggested underlying mechanisms were ascertainment bias (e.g. misdiagnosis and cultural bias in assessments) discussed in four studies,^17^^,^^24^^,^^27^^,^^34^ selective mating (i.e. fathers with autistic traits seeking mates abroad) in four studies^25^^,^^30^^,^^41^^,^^43^ and selective migration in two studies.^16^^,^^29^

#### Decreased risk of any ASD and ASD – ID

In all, 14 studies suggested ascertainment bias, including under-diagnosis and misdiagnosis/misclassification, as an underlying mechanism of the decreased risk of a diagnosis of any ASD and ASD – ID in migrants, descendants of migrants or ethnic minorities.^16^^,^^17^^,^^19^^,^^20^^,^^24^^,^^28^^,^^33^^,^^34^^,^^39^^,^^42^^,^^44–47^ Under-diagnosis was suggested to depend on lower health-care utilization (e.g. lack of access to health-care services, lack of awareness of ASD or service availability, fear of stigma, cultural barriers, language barriers and no medical insurance or low income) or health-care disparities (e.g. differences in cognitive testing practices, differential referral patterns to specialists, difficulties in understanding the meaning of culturally shaped symptoms and communication problem). Apart from that, four studies discussed about selective migration,^27^^,^^28^^,^^35^^,^^39^ one study random error due to small sample sizes,^42^ one study genetic factors^33^ and one study true decrease in risk (i.e. low initial rates in original country and environmental factors in new country).^39^

#### Increased risk of severe/profound ID

Four studies suggested genetic factors, including consanguinity and genetic abnormalities, as underlying mechanisms of the increased risk of severe or profound ID in migrants, descendants of migrants or ethnic minorities.^36^^,^^38^^,^^48^^,^^49^ In addition, misclassification of severity was discussed in two studies,^24^^,^^48^ environmental factors in one study^48^ and inequalities in access to maternal health care in one study.^48^

#### Decreased risk of mild ID

Three studies suggested ascertainment bias, including under-diagnosis and misclassification of severity as underlying mechanisms of the decreased risk of mild ID in migrants, descendants of migrants or ethnic minorities.^24^^,^^48^^,^^50^ The authors discussed lower health-care utilization and health-care disparities as the causes for under-diagnosis, similarly to the studies on the decreased risk of any ASD and ASD – ID described above.

## Discussion

This systematic review investigated the association between migration or ethnic minority status and ASD and ID and summarized hypotheses for underlying mechanisms. The results indicated an increased risk of ASD + ID and a decreased risk of ASD – ID in migrants, descendants of migrants and ethnic minorities. Data on ID were scarce but suggested an increased risk of severe or profound ID and a decreased risk of mild ID in some ethnic minorities. Risks for any ASD or ASD + ID appeared more pronounced with maternal when compared with paternal migration status, with origin in low-income countries and among descendants of migrants when compared with migrants. Suggested mechanisms explaining the increased risks of ASD + ID and severe/profound ID included environmental factors acting *in utero* and genetic factors (including consanguinity), while ascertainment bias was proposed to account for the lowered risks of diagnosed ASD – ID and mild ID.

### Risks of ASD and ID

The results for ASD + ID and ASD – ID were consistent with the previous systematic reviews studying ASD^13^^,^^22^ and autistic disorder.^21^ When a co-occurrence of ID was not considered, studies showed both increased and decreased risks of ASD. This variation may depend on the case-mix in the study populations, i.e. the proportion of ASD cases with ID. For example, a study included in our review as an investigation of ‘Any ASD’ that had a large proportion of a severe ASD had a similar result as studies for ASD + ID.^25^ This suggests that the association between migration or ethnic minority status and ASD needs to be studied separately for ASD + ID and ASD – ID.

There were only few studies on the association between migration or ethnic minority status and ID, and most of them were not of high-quality. Especially, studies on ID without ASD were scant. Further studies that compare risks between ASD + ID and ID without ASD are needed in order to draw a conclusion for whether the increases in risks are specifically linked to ASD + ID or reflect a general association with ID. In addition, the notable differences in the risks of ID by severity should be examined further.

### Migration-related or ethnically determined factors

Migration-related factors acting *in utero* and/or associated with origin in low-income countries may be important drivers of ASD + ID risk in migrants and descendants of migrants. First, the observed stronger association for maternal migration status suggest that ethnically determined genetic factors may not be as important as environmental factors operating *in utero* for ASD + ID. Yet, differences in parental origin was not fully accounted for and may explain differential findings for maternal and paternal migration. This discrepancy may, however, also be due to maternal migration-related factors acting *in utero*. Secondly, the generally stronger associations with origin in low-income countries in different continents suggest that ASD and ID may not be associated with a specific country of extraction or a specific ethnicity. Migration-related factors that are generally associated with origin in low-income countries may therefore be of higher relevance. Thirdly, the results of higher risks in descendants of migrants, especially when maternal migration occurs around birth of the index child, suggest that factors related to the migration process and acting *in utero* are possibly associated with ASD + ID.

### Potential underlying mechanisms

#### Increased risk of ASD + ID and severe/profound ID

There are many studies on the association between environmental factors *in utero* and ASD and ID. Some of them have suggested that prenatal maternal stress is possibly associated with ASD and autistic traits in the offspring and ASD-related symptom severity.^51–55^ A history of intrauterine exposure to toxins, including alcohol, cocaine, marijuana, mercury and pollutants, has been associated with lower IQ scores and ASD.^56^^,^^57^ In addition, ASD and ID has been linked to maternal infection and fever during pregnancy.^58–62^ There are also reports of lower levels of prenatal and neonatal vitamin D among individuals with ASD.^63^ It has been proposed that these factors affect maternal immune activation and neuro-inflammation, which may contribute to the development of a subset of ASD.^64^^,^^65^ It is, however, not possible to infer causality based on the evidence from the studies. Moreover, it remains to be determined to what extent migrants, descendants of migrants and ethnic minorities are exposed to these factors *in utero*.

Consanguinity is another suggested underlying mechanism. For example, in one study from the UK, the rate of consanguinity in a Pakistani population was around 60%, and this population had an increased risk of severe/profound ID.^38^ In many communities in the Middle East, Central and South Asia and Africa, favouring strict intracommunity marriage, 20 to more than 60% of all marriages are between first cousins.^66^ Consanguineous mating may lead to novel mutations in genes known to be associated with ID.^67^ As consanguinity is largely preventable, further confirmative studies on its role in the development of ASD and ID are needed.

#### Decreased risk of ASD – ID and mild ID

This systematic review supports the notion that the ASD – ID and the mild ID in migrants, descendants of migrants and ethnic minorities may be under-diagnosed. Previous studies have shown that these groups face a variety of barriers such as stigma,^68^ lack of knowledge and differential perceptions of ASD,^69^^,^^70^ differential referral patterns^71^ and delays in diagnosis.^70^ However, this issue is poorly investigated and further research is urgently needed.

### Strengths and limitations

This study has several strengths. To the best of our knowledge, this is the first systematic review on the association between migration or ethnic minority status and ID. In addition, there has not been any review on migration and ethnic minority status focusing on ASD with and without co-occurrence of ID. We also believe that our structured comparisons of migrants, descendants of migrants and ethnic minorities are strengths.

However, there are also some limitations. First, there are possibly relevant studies that were not included in the review, because they were not in the databases used for the search. Secondly, diagnostic criteria for ASD and ID have changed over time, and diagnostic practices, including the practice of diagnosing co-morbidity of other developing disorders in individuals with ASD or ID, vary between countries or regions. This led to a heterogeneity in a case ascertainment, hampering proper categorization and comparison between the included studies. For example, autistic disorder, which usually implies a more severe type of ASD, may present without ID, but it was categorized as the ASD + ID category, and Asperger syndrome, which is not characterized by a clinically significant delay in cognitive development,^72^ was categorized as ASD – ID. We believe, however, that this should not affect the overall results and conclusions of this review. Thirdly, many of the included studies were at high risk of bias. For example, there could be a misclassification of exposures in the studies that defined migration status based on maternal country of birth.

### Significance

ASD and ID place a heavy burden on affected individuals, their family and society. Both conditions often impact considerably on quality of life and welfare, and are associated with increased mortality and morbidity.^73^ Furthermore, the early onset and generally lifelong impairments results in a high societal cost.^74^ Improved understanding of any link between migration or ethnic minority status and ASD and ID may not only provide clues to modifiable causes of ASD and ID, but also provide vital information for the planning of health and education services in countries having a continued large influx of international migrants over foreseeable time. Moreover, findings of underlying mechanisms may ultimately lead to the development of preventive interventions, which may substantially reduce the burden of disease.

### Future studies

This systematic review suggests several future areas for research. First, this review emphasizes the need of studies on the association between migration or ethnic minority status and ID, with a focus on ID with and without ASD. In addition, it is also important to study on the association with other neurodevelopmental disorders such as attention-deficit hyperactivity disorder. Secondly, further research is needed in order to examine the hypothesis suggested by this review that migration-related factors acting *in utero* and/or associated with origin in low-income countries may be important drivers of ASD + ID risk. In addition, the role of consanguinity in the development of ASD and ID also needs to be studied. Thirdly, it is important to determine whether the decreased risks of ASD – ID and mild ID in migrants, descendants of migrants and ethnic minority status is a result of under-diagnosis, in order to reduce social inequality and improve health-care services.

## Supplementary data

Supplementary data are available at *EURPUB* online.

## Funding

This work was supported by the Swedish Research Council (grant number 2015-02529).

*Conflicts of interest*: None declared.

**Key points**

- The summarized evidence indicated an increased risk of ASD + ID and a decreased risk of ASD – ID in migrants, descendants of migrants, and ethnic minorities.
- The results suggested that migration-related factors acting *in utero* and/or associated with origin in low-income countries may be important in the ASD + ID aetiology.
- Further studies investigating whether ascertainment bias is associated with the lowered risks of ASD – ID are needed in order to reduce social inequality and improve health-care services.
- Data on ID were scarce, which emphasized the need of studies on the association between migration or ethnic minority status and ID.

## Supplementary Material

ckaa108_Supplementary_Data
