# Supplementary material for: Migration or ethnic minority status and risk of autism spectrum disorders and intellectual disability: systematic review
Source: Eur J Public Health. 2020 Oct 13;31(2):304–12. doi: 10.1093/eurpub/ckaa108 (PMC8071599; doi:10.1093/eurpub/ckaa108)
Supplement: ckaa108_Supplementary_Data [file ckaa108_supplementary_data.docx]

**Reference (continued)**

41. Gillberg IC, Gillberg C. Autism in immigrants: a population-based study from Swedish rural and urban areas. J Intellect Disabil Res. 1996;40(Pt 1):24-31.

42. Pedersen A, Pettygrove S, Meaney FJ, Mancilla K, Gotschall K, Kessler DB, et al. Prevalence of autism spectrum disorders in hispanic and non-hispanic white children. Pediatrics. 2012;129(3):e629-e35.

43. Williams K, Helmer M, Duncan GW, Peat JK, Mellis CM. Perinatal and maternal risk factors for autism spectrum disorders in New South Wales, Australia. Child Care Health Dev. 2008;34(2):249-56.

44. Durkin MS, Maenner MJ, Baio J, Christensen D, Daniels J, Fitzgerald R, et al. Autism Spectrum Disorder Among US Children (2002-2010): Socioeconomic, Racial, and Ethnic Disparities. Am J Public Health. 2017;107(11):1818-26.

45. Mehta NK, Lee H, Ylitalo KR. Child health in the United States: recent trends in racial/ethnic disparities. Soc Sci Med. 2013;95:6-15.

46. Raz R, Weisskopf MG, Davidovitch M, Pinto O, Levine H. Differences in Autism Spectrum Disorders Incidence by Sub-Populations in Israel 1992–2009: A Total Population Study. J Autism Dev Disord. 2015;45(4):1062-9.

47. Davidovitch M, Hemo B, Manning-Courtney P, Fombonne E. Prevalence and incidence of autism spectrum disorder in an Israeli population. J Autism Dev Disord. 2013;43(4):785-93.

48. Emerson E, Azmi S, Hatton C, Caine A, Parrott R, Wolstenholme J. Is there an increased prevalence of severe learning disabilities among british asians? Ethn Health. 1997;2(4):317-21.

49. Fernell E. Aetiological factors and prevalence of severe mental retardation in children in a Swedish municipality: The possible role of consanguinity. Dev Med Child Neurol. 1998;40(9):608-11.

50. McGrother CW, Bhaumik S, Thorp CF, Watson JM, Taub NA. Prevalence, morbidity and service need among South Asian and white adults with intellectual disability in Leicestershire, UK. J Intellect Disabil Res. 2002;46(4):299-309.

51. Class QA, Abel KM, Khashan AS, Rickert ME, Dalman C, Larsson H, et al. Offspring psychopathology following preconception, prenatal and postnatal maternal bereavement stress. Psychol Med. 2014;44(1):71-84.

52. Say GN, Karabekiroglu K, Babadagi Z, Yuce M. Maternal stress and perinatal features in autism and attention deficit/hyperactivity disorder. Pediatr Int. 2016;58(4):265-9.

53. Ronald A, Pennell CE, Whitehouse AJ. Prenatal Maternal Stress Associated with ADHD and Autistic Traits in early Childhood. Front Psychol. 2010;1:223.

54. Walder DJ, Laplante DP, Sousa-Pires A, Veru F, Brunet A, King S. Prenatal maternal stress predicts autism traits in 6(1/2) year-old children: Project Ice Storm. Psychiatry Res. 2014;219(2):353-60.

55. Varcin KJ, Alvares GA, Uljarevic M, Whitehouse AJO. Prenatal maternal stress events and phenotypic outcomes in Autism Spectrum Disorder. Autism Res. 2017;10(11):1866-77.

56. Perrone-McGovern K, Simon-Dack S, Niccolai L. Prenatal and Perinatal Factors Related to Autism, IQ, and Adaptive Functioning. J Genet Psychol. 2015;176(1-2):1-10.

57. Leslie K, Koger S. A Significant Factor in Autism: Methyl Mercury Induced Oxidative Stress in Genetically Susceptible Individuals. J Dev Phys Disabil. 2011;23(4):313-24.

58. Brucato M, Ladd-Acosta C, Li M, Caruso D, Hong X, Kaczaniuk J, et al. Prenatal exposure to fever is associated with autism spectrum disorder in the boston birth cohort. Autism Res. 2017;10(11):1878-90.

59. Fang SY, Wang S, Huang N, Yeh HH, Chen CY. Prenatal Infection and Autism Spectrum Disorders in Childhood: A Population-Based Case-Control Study in Taiwan. Paediatr Perinat Epidemiol. 2015;29(4):307-16.

60. Flinkkila E, Keski-Rahkonen A, Marttunen M, Raevuori A. Prenatal Inflammation, Infections and Mental Disorders. Psychopathology. 2016;49(5):317-33.

61. Haber H, Xing G, Walker C. Prenatal infections and risk of autism, intellectual disability and/or epilepsy. Am J Obstet Gynecol. 2016;215(6):S824.

62. Hornig M, Bresnahan MA, Che X, Schultz AF, Ukaigwe JE, Eddy ML, et al. Prenatal fever and autism risk. Mol Psychiatry. 2018;23(3):759-66.

63. Mazahery H, Camargo CA, Jr., Conlon C, Beck KL, Kruger MC, von Hurst PR. Vitamin D and Autism Spectrum Disorder: A Literature Review. Nutrients. 2016;8(4):236.

64. Bilbo SD, Block CL, Bolton JL, Hanamsagar R, Tran PK. Beyond infection - Maternal immune activation by environmental factors, microglial development, and relevance for autism spectrum disorders. Exp Neurol. 2018;299(Pt A):241-51.

65. Udagawa J, Hino K. Impact of Maternal Stress in Pregnancy on Brain Function of the Offspring. Nihon Eiseigaku Zasshi. 2016;71(3):188-94.

66. Britannica Academic [Internet]. 2019. Available from: https://academic-eb-com.proxy.kib.ki.se/levels/collegiate.

67. Alkhateeb AM, Aburahma SK, Habbab W, Thompson IR. Novel mutations in WWOX, RARS2, and C10orf2 genes in consanguineous Arab families with intellectual disability. Metab Brain Dis. 2016;31(4):901-7.

68. Ellen Selman L, Fox F, Aabe N, Turner K, Rai D, Redwood S. 'You are labelled by your children's disability' - A community-based, participatory study of stigma among Somali parents of children with autism living in the United Kingdom. Ethn Health. 2018;23(7):781-96.

69. Fox F, Aabe N, Turner K, Redwood S, Rai D. "It was like walking without knowing where I was going": A Qualitative Study of Autism in a UK Somali Migrant Community. J Autism Dev Disord. 2017;47(2):305-15.

70. Magana S, Lopez K, Aguinaga A, Morton H. Access to diagnosis and treatment services among latino children with autism spectrum disorders. Intellect Dev Disabil. 2013;51(3):141-53.

71. Begeer S, Bouk SE, Boussaid W, Terwogt MM, Koot HM. Underdiagnosis and referral bias of autism in ethnic minorities. J Autism Dev Disord. 2009;39(1):142-8.

72. Association AP. Diagnostic and Statistical Manual of Mental Disorders, Fourth Edition. Arlington: VA: American Psychiatric Publishing; 1994.

73. Duchan E, Patel DR. Epidemiology of autism spectrum disorders. Pediatr Clin North Am. 2012;59(1):27-43, ix-x.

74. Buescher AV, Cidav Z, Knapp M, Mandell DS. Costs of autism spectrum disorders in the United Kingdom and the United States. JAMA pediatr. 2014;168(8):721-8.

75. Kogan MD, Strickland BB, Blumberg SJ, Singh GK, Perrin JM, van Dyck PC. A national profile of the health care experiences and family impact of autism spectrum disorder among children in the United States, 2005-2006. Pediatrics. 2008;122(6):e1149.

76. Maimburg RD, Væth M. Perinatal risk factors and infantile autism. Acta Psychiatr Scand. 2006;114(4):257-64.

77. Wing L. Childhood autism and social class: a question of selection? Br J Psychiatry. 1980;137:410.

78. Yeargin-Allsopp M, Rice C, Karapurkar T, Doernberg N, Boyle C, Murphy C. Prevalence of autism in a US metropolitan area. JAMA. 2003;289(1):49-55.

**Fig. S1 Venn diagram for migrants and ethnic minorities in a host country population**

* including people migrating as a foster and born in a host country

| **Table S1. Search Strategy** | | |
| --- | --- | --- |
| (1) Ovid MEDLINE and Epub Ahead of Print, In-Process & Other Non-Indexed Citations and Daily | | |
|  | **Search terms** | **Items found** |
| 1 | exp Autism spectrum disorder/ | 24513 |
| 2 | autism.ti,ab,kf. | 36493 |
| 3 | Asperger syndrome.ti,ab,kf. | 1010 |
| 4 | (Pervasive developmental disorder not otherwise specified).ti,ab,kf. | 796 |
| 5 | Infantile autism.ti,ab,kf. | 798 |
| 6 | Autistic disorder*.ti,ab,kf. | 1601 |
| 7 | or/1-6 | 42528 |
| 8 | exp Intellectual disability/ | 91265 |
| 9 | Mental retardation.ti,ab,kf. | 26705 |
| 10 | Learning disability.ti,ab,kf. | 2876 |
| 11 | or/8-10 | 104663 |
| 12 | 7 or 11 | 141987 |
| 13 | exp "Emigration and Immigration"/ | 24470 |
| 14 | exp "Transients and Migrants"/ | 10311 |
| 15 | exp Refugees/ | 8820 |
| 16 | Migration.ti,ab,kf. | 222734 |
| 17 | Immigration.ti,ab,kf. | 10464 |
| 18 | Emigration.ti,ab,kf. | 4750 |
| 19 | Migrant*.ti,ab,kf. | 17117 |
| 20 | Immigrant*.ti,ab,kf. | 22666 |
| 21 | Refugee*.ti,ab,kf. | 9113 |
| 22 | Asylum Seeker*.ti,ab,kf. | 1310 |
| 23 | or/13-22 | 277149 |
| 24 | exp Ethnology/ | 1577 |
| 25 | exp Ethnic Groups/ | 140713 |
| 26 | exp Minority Groups/ | 12615 |
| 27 | Ethnicity.ti,ab,kf. | 57519 |
| 28 | Ethnic minorit*.ti,ab,kf. | 9463 |
| 29 | Ethnic group*.ti,ab,kf. | 32614 |
| 30 | Race.ti,ab,kf. | 92610 |
| 31 | or/24-30 | 269018 |
| 32 | 23 or 31 | 532805 |
| 33 | 12 and 32 | 2740 |
| 34 | limit 33 to human | 2400 |
| 35 | limit 34 to abstracts | 2075 |
|  | | |
| (2) PsycINFO | | |
|  | **Search terms** | **Items found** |
| 1 | exp Autism spectrum disorders/ | 38895 |
| 2 | autism.ti,ab,id. | 41433 |
| 3 | Asperger syndrome.ti,ab,id. | 1717 |
| 4 | (Pervasive developmental disorder not otherwise specified).ti,ab,id. | 1084 |
| 5 | Infantile autism.ti,ab,id. | 958 |
| 6 | Autistic disorder*.ti,ab,id. | 1782 |
| 7 | or/1-6 | 46669 |
| 8 | exp Intellectual development disorder/ | 43234 |
| 9 | Mental retardation.ti,ab,id. | 28661 |
| 10 | Learning disability.ti,ab,id. | 5303 |
| 11 | or/8-10 | 53809 |
| 12 | 7 or 11 | 96594 |
| 13 | exp Human migration/ | 11095 |
| 14 | exp Immigration/ | 20444 |
| 15 | Migration.ti,ab,id. | 16453 |
| 16 | Immigration.ti,ab,id. | 10462 |
| 17 | Emigration.ti,ab,id. | 1091 |
| 18 | Migrant*.ti,ab,id. | 8863 |
| 19 | Immigrant*.ti,ab,id. | 24521 |
| 20 | Refugee*.ti,ab,id. | 7346 |
| 21 | Asylum Seeker*.ti,ab,id. | 1124 |
| 22 | or/13-21 | 53207 |
| 23 | exp Ethnology/ | 1973 |
| 24 | exp "Racial and Ethnic Groups"/ | 118854 |
| 25 | exp Minority Groups/ | 13978 |
| 26 | Ethnicity.ti,ab,id. | 36799 |
| 27 | Ethnic minorit*.ti,ab,id. | 10561 |
| 28 | Ethnic group*.ti,ab,id. | 16047 |
| 29 | Race.ti,ab,id. | 60938 |
| 30 | or/23-29 | 198363 |
| 31 | 22 or 30 | 235724 |
| 32 | 12 and 31 | 1970 |
| 33 | limit 32 to human | 1847 |
| 34 | limit 33 to abstracts | 1730 |
|  | | |
| (3) Embase | | |
|  | **Search terms** | **Items found** |
| #1 | 'autism'/exp | 60,737 |
| #2 | autism:ti,ab,kw | 47,428 |
| #3 | 'asperger syndrome':ti,ab,kw | 1,543 |
| #4 | 'pervasive developmental disorder not otherwise specified':ti,ab,kw | 460 |
| #5 | 'infantile autism':ti,ab,kw | 988 |
| #6 | 'autistic disorder*':ti,ab,kw | 2,430 |
| #7 | #1 OR #2 OR #3 OR #4 OR #5 OR #6 | 66,121 |
| #8 | 'mental deficiency'/exp | 141,342 |
| #9 | 'intellectual disability':ti,ab,kw | 16,004 |
| #10 | 'mental retardation':ti,ab,kw | 35,138 |
| #11 | 'learning disability':ti,ab,kw | 4,121 |
| #12 | #8 OR #9 OR #10 OR #11 | 164,862 |
| #13 | #7 OR #12 | 219,298 |
| #14 | 'migration'/exp | 43,274 |
| #15 | 'migrant'/exp | 32,171 |
| #16 | migration:ti,ab,kw | 277,984 |
| #17 | immigration:ti,ab,kw | 11,586 |
| #18 | emigration:ti,ab,kw | 5,036 |
| #19 | migrant*:ti,ab,kw | 16,562 |
| #20 | immigrant*:ti,ab,kw | 25,961 |
| #21 | refugee*:ti,ab,kw | 9,731 |
| #22 | 'asylum seeker*':ti,ab,kw | 1,521 |
| #23 | #14 OR #15 OR #16 OR #17 OR #18 OR #19 OR #20 OR #21 OR #22 | 349,905 |
| #24 | 'ethnic or racial aspects'/exp | 231,091 |
| #25 | 'ethnic group'/exp | 157,991 |
| #26 | 'ethnology'/exp | 74,210 |
| #27 | ethnicity:ti,ab,kw | 85,810 |
| #28 | 'ethnic minorit*':ti,ab,kw | 11,664 |
| #29 | 'ethnic group*':ti,ab,kw | 38,489 |
| #30 | race:ti,ab,kw | 136,986 |
| #31 | #24 OR #25 OR #26 OR #27 OR #28 OR #29 OR #30 | 523,777 |
| #32 | #23 OR #31 | 848,359 |
| #33 | #13 AND #32 | 6,621 |
| #34 | #13 AND #32 AND [humans]/lim | 5,851 |
| #35 | #13 AND #32 AND [humans]/lim AND [abstracts]/lim | 5,273 |

| **Table S2. Definitions for exposures** | |
| --- | --- |
| Exposures | Bases on definitions in the included studies |
| Migration | - Maternal country of birth - Maternal region of birth - Whether mother is born in the UK or not - Paternal region of birth - Maternal and paternal region of birth - Maternal and paternal citizenship - Migration status of at least one parent - Children´s country of birth - Immigrant (= foreign-born children with both immigrant parents or US-born children with one or both immigrant parents) or native-born (= non-immigrant or US-born parents) - Somali (= children born in Somalia or Sweden, with both parents born abroad and at least one parent coming from Somali) or non-Somali |
| Ethnic minority status | - Children´s ethnicity - Maternal ethnicity |

| **Table S3. Definitions for outcomes** | |
| --- | --- |
| Outcomes | Definitions in the included studies |
| Any ASD | - ASD including autistic disorder (DSM-IV/DSM-IV-TR/ICD-9: 299.00-299.01 or ICD-10: F84.0), PDD-NOS (DSM-IV/DSM-IV-TR/ICD-9: 299.90-299.91 or ICD-10: F84.9), and Asperger disorder (DSM-IV/DSM-IV-TR/ICD-9: 299.80-299.81 or ICD-10: F84.5) - ASD according to a formal statement of special educational needs - PDDs including autism, Rett’s disorder, childhood disintegrative disorder, Asperger’s syndrome, and PDD-NOS - Full syndrome autism defined by the California Department of Developmental Services - Autism (nuclear and non nuclear) - Yes on question “Ever diagnosed with autism?” - Yes on questions “Were you ever told by a doctor or other health care provider that [CHILD] had autism, Asperger’s disorder, pervasive developmental disorder, or other autism spectrum disorder?” and “Does [CHILD] currently have autism or ASD?” - Yes on question “Does your child currently have autism or ASD?” |
| ASD–ID | - ASD including autistic disorder (DSM-IV/DSM-IV-TR/ICD-9: 299.00-299.01 or ICD-10: F84.0), PDD-NOS (DSM-IV/DSM-IV-TR/ICD-9: 299.90-299.91 or ICD-10: F84.9), and Asperger disorder (DSM-IV/DSM-IV-TR/ICD-9: 299.80-299.81 or ICD-10: F84.5) without a comorbid ID (defined as IQ<70) - Autism diagnosis without a comorbid ID (defined as IQ<70) recorded in Swedish Healthcare registers - Asperger syndrome (DSM-IV: 299.80 or ICD-10: F84.5) |
| ASD+ID | - ASD including autistic disorder (DSM-IV/DSM-IV-TR/ICD-9: 299.00-299.01 or ICD-10: F84.0), PDD-NOS (DSM-IV/DSM-IV-TR/ICD-9: 299.90-299.91 or ICD-10: F84.9), and Asperger disorder (DSM-IV/DSM-IV-TR/ICD-9: 299.80-299.81 or ICD-10: F84.5) with a comorbid ID (defined as IQ < 70) - Autism diagnosis with a comorbid ID (IQ <70) recorded in Swedish Healthcare registers - Autistic disorder (DSM-IV/ICD-9-CM: 299.00) and MR (DSM-IV/ICD-9-CM: 317.00, mild; 318.00, moderate; 318.10, severe; 318.20, profound; 319.00, MR unspecified) - Autistic disorder (DSM-III/DSM-IV/ICD-9-CM: 299.00) - Infantile autism (DSM-III/ICD-8/ICD-9:299.00 or ICD-10: F84.0) - Childhood autism (ICD-9: 299.00 or ICD-10: F84.0) - Childhood autism (ICD-10: F84.0) or atypical autism (ICD-10: F84.1) - Autistic disorder (DSM-IV: 299.00) or childhood autism (ICD-10: F84.0) - Autism or PDD-NOS (all had learning disabilities) - ASD except milder forms such as Asperger’s syndrome |
| Any ID | - ID defined as IQ<70 - ID defined by "Heber 1959" and WHO 1992 - Learning difficulties according to a formal statement of special educational needs (UK study) - Learning disabilities according to health or educational services/ project staff/ a pre-existing Case Register (UK study) - Severe learning disability according to DSM-IV / defined as IQ<50 - Idiopathic MR without autism or cerebral palsy, defined by the California Department of Developmental Services. Mild (IQ 50-70), severe (IQ < 50), or unspecified according to ICD-9 - Isolated MR (without other neurological conditions): mild (IQ 50-70) and severe (IQ <50) |
| ASD = autism spectrum disorder; ID = intellectual disability; PDDs = pervasive developmental disorders; PDD-NOS = pervasive developmental disorder-not otherwise specified; MR = mental retardation; DSM = Diagnostic and Statistical Manual of Mental Disorders; ICD = International Classification of Diseases | |

| **Table S4. Any ASD** | | | | | | | | |
| --- | --- | --- | --- | --- | --- | --- | --- | --- |
| **First author**  (publication data**)** | **Location** | **Design**  (Source of data, Population,  Sample size and description)  **Study quality with NOS** | **Case ascertainment** | **Exposure** (migration: either migrants or descendants of migrants) | **Results** | | | **Adjustment factor** |
| Durkin (2017) | 11 sites,  USA | Cross-sectional  (Multiple-source surveillance (ADDM),  Population estimated using a census,  Children ages 8 in 2002, 2006, 2008 and 2010 (n = 1,308,641),  13,396 cases)  NOS: 3 | Autistic Disorder, PDD-NOS, and Asperger Disorder DSM-IV-TR/ | Ethnic minority status | *Number of cases/ total-number:*  2002:  Non-Hispanic White 1,522/ 226,134  Non-Hispanic Black 476/ 81,402  Hispanic 192/ 49,696  2006, 2008, and 2010:  Non-Hispanic White 6,660/ 503,812  Non-Hispanic Black 2,055/ 185,648  Hispanic 1,341/ 166,898  *Prevalence (95% CI):*  2002:  Non-Hispanic White 6.7 per 1000 (6.4-7.0)  Non-Hispanic Black 5.9 per 1000 (5.4-6.5)  Hispanic **3.9 per 1000 (3.4-4.5)**  2006, 2008, and 2010:  Non-Hispanic White 13.2 per 1000 (12.9-13.5)  Non-Hispanic Black **11.1 per 1000 (10.6-11.6)**  Hispanic **8.0 per 1000 (7.6-8.4)**  *Prevalence ratio (95% CI):*  2002:  Non-Hispanic White/Non-Hispanic Black **1.15 (1.04-1.38)**  Non-Hispanic White/Hispanic **1.74 (1.50-2.02)**  2006, 2008, and 2010:  Non-Hispanic White/Non-Hispanic Black **1.19 (1.14-1.25)**  Non-Hispanic White/Hispanic **1.65 (1.56-1.74)** | | | - |
| Hewitt  (2016) | Minneapolis,  USA | Cross-sectional  (Multiple source (MSASDPP),  Compared to population estimated using a census,  Children ages 7-9 (n=12,329),  255 cases)  NOS: 4 | Autistic Disorder, PDD-NOS (including atypical autism), and Asperger Disorder DSM-IV-TR | Ethnic minority status | *Number of cases/ total-number:*  White 120/ 4,336  Black (non-Somali) 53/ 3,312  Somali 31/ 1,007  Hispanic 30/ 2,399  *Prevalence:*  White 27.7 per 1000  Black (non-Somali) **16.0 per 1000** p<0.05  Somali 30.8 per 1000  Hispanic **12.5 per 1000** p<0.05 | | | - |
| Raz (2015) | Israel | Retrospective cohort  (Register,  Children born 1992-2009 (n= 2,431,649),  9,109 cases)  NOS: 5 | ASD DSM-IV-TR | Ethnic minority status:  Father’s status in ID for IA, and father’s or siblings’ studies at a religious college (‘‘Yeshiva’’) for UOJ | *Number of cases/ total-number:*  General population (total population, excluding IA and UOJ) 7,469/ 1,326,303  Israeli Arabs (IA) 584/ 674,679  Ultra-orthodox Jews (UOJ) 1,056/ 430,667  *Prevalence (calculated from Table 1):*  General population 5.6 per 1000  Israeli Arabs (IA) **0.9 per 1000**  Ultra-orthodox Jews (UOJ) **2.5 per 1000** | | | - |
| Davidovitch (2013) | Israel | Retrospective cohort (Healthcare service,  children age 1-12 (n=423,524),  2,034 cases diagnosed before 2010)  NOS: 7 | Autistic disorder,  Asperger’s syndrome, and PDD-NOS DSM-IV | Ethnic minority status:  Place of residence (e.g. Umm el-Fahm populated exclusively by Israeli Arabs, and Modi’in Illit by ultra-orthodox Jews) | *Number of cases/ total-number:*  General population 1,830/ 330,159  Israeli Arab in rural settlements 30/ 26,077  Ultra-orthodox Jews 174/ 67,288  *Prevalence (95% CI):*  General population 5.5 per 1000 (5.25-5.75)  Israeli Arab in rural settlements **1.2 per 1000 (0.78-1.62)**  Ultra-orthodox Jews **2.6 per 1000 (2.22-2.98)**  For 5 year olds born in 2002 through 2005, a statistically significant increase in annual cumulative incidence was seen for the general population, but not for the minority populations. | | | - |
| Mehta  (2013) | USA | Cross-sectional  (Survey (NHIS),  Population estimated using  the pooled 1998-2009 data,  Children age 0-16 (n=286,232),  No description for number of cases)  NOS: 3 | Question: ever diagnosed with autism? (Yes/No) | Ethnic minority status | *Number of cases/ total-number:*  Non-Hispanic white -/ 132,710  Non-Hispanic black -/ 45,704  Hispanic -/ 85,766  Non-Hispanic Asian -/ 10,073  Non-Hispanic other -/ 4,177  *Prevalence %:*  Non-Hispanic white 0.61  Non-Hispanic black 0.50  Hispanic 0.37  Non-Hispanic Asian 0.32  Non-Hispanic other 1.24  Large increases in the prevalence of autism between 1998 and 2009, with proportionate and absolute increases being larger for whites compared to blacks and Hispanics. | | | - |
| Singh  (2013) | USA | Cross-sectional  (Survey (NSCH),  Children aged 0-17 (n=91 532),  No description for numbers of cases, aged 3-17)  NOS: 4 | Questions: ‘‘Were you ever told by a doctor or other health care provider that [CHILD] had autism, Asperger’s disorder, pervasive developmental disorder, or other autism spectrum disorder?’’  ‘‘Does [CHILD] currently have autism or ASD?’’ | Both migration and  ethnic minority status:  Children’s immigration status (Immigrant = foreign-born children with both immigrant parents or US-born children with one or both immigrant parents.  Native-born = non-immigrant or US-born parents) and race/ethnicity | *Number of cases/ total-number:*  Hispanic -Immigrant -/ 5,364  -Native-born -/ 6,149  Non-Hispanic white -Immigrant -/ 3,775  -Native-born -/ 57,617  Non-Hispanic black -Immigrant -/ 738  -Native-born -/ 8,149  Asian -Immigrant -/ 1,555  -Native-born -/ 760  *Prevalence:* p<0.05  Hispanic -Immigrant 0.71%  -Native-born 1.40%  Non-Hispanic white -Immigrant 1.07%  -Native-born 1.26%  Non-Hispanic black -Immigrant 0.69%  -Native-born 0.60%  Asian -Immigrant 0.28%  -Native-born 0.85%  *Adjusted OR (95% CI):*  Hispanic -Immigrant 0.50 (0.21-1.18)  -Native-born 1.08 (0.54-2.15)  Non-Hispanic white -Immigrant 0.87 (0.48-1.56)  -Native-born 1.00  Non-Hispanic black -Immigrant 0.55 (0.18-1.74)  -Native-born **0.43 (0.23-0.82)**  Asian -Immigrant **0.22 (0.10-0.51)**  -Native-born 0.65 (0.19-2.25)  Other -Immigrant 0.57 (0.27-1.22)  -Native-born 1.22 (0.57-2.59) | | | Child’s age, sex, household composition,  metropolitan/non-metropolitan residence, household poverty, and  education level |
| Van der Ven  (2013) | Netherlands | Retrospective cohort  (Register,  Children born 1998-2007 (n = 106 953),  518 cases)  NOS: 8 | Autistic disorder (DSM-IV code 299.00), Asperger syndrome (299.80) or PDD-NOS (299.80) | Migration (descendants of migrants):  Maternal country of birth  Paternal migrant status  (n = 37) | *Number of cases/ total-number:*  Netherlands 420/ 80,354  Developing countries 69/ 19,948  Developed countries 29/ 6,651  *Adjusted rate ratio (95% CI):*  Netherlands 1.0  Developing countries  All **0.6 (0.5-0.9)**  Turkey 0.8 (0.5-1.5)  Morocco **0.3 (0.2-0.6)**  Suriname and Dutch Antilles 1.2 (0.7-2.1)  Other 0.8 (0.5-1.3)  Developed countries  All 0.9 (0.6-1.3)  *Rate ratio (95% CI):*  Native Dutch 1.0  A Dutch mother and a foreign father 1.2 (0.8-1.8) | | | Gender and paternal age |
| Emerson (2012) | UK | Cross-sectional  (School census,  Children aged 7-15 in 2008 (n=5,180,550),  case 0.6% of the total sample)  NOS: 4 | autistic spectrum disorder according to a formal statement of special educational needs | Ethnic minority status | *Number of cases/ total-number:*  African -/ 2.5% of the total sample  Other Asian Background -/ 1.1% of the total sample  Other Black background -/ 0.5% of the total sample  Other ethnic group -/ 1.1% of the total sample  Other Mixed Background -/ 1.2% of the total sample  Other White background -/ 3.1% of the total sample  Bangladeshi -/ 1.3% of the total sample  Caribbean -/ 1.4% of the total sample  Chinese -/ 0.4% of the total sample  Gypsy/Romany -/ 0.1% of the total sample  Indian -/ 2.3% of the total sample  Irish -/ 0.4% of the total sample  Pakistani -/ 3.1% of the total sample  Traveller of Irish heritage -/ 0.1% of the total sample  White and Asian -/ 0.7% of the total sample  White and Black African -/ 0.3% of the total sample  White and Black Caribbean -/ 1.2% of the total sample  White British -/ 78.2% of the total sample  *OR (95% CI):*  African 0.10 (0.88-1.02)  Other Asian Background **0.58 (0.51-0.66)** p<0.001  Other Black background 1.17 (1.03-1.34)  Other ethnic group **0.50 (0.43-0.57)** p<0.001  Other Mixed Background 1.19 (1.02-1.23)  Other White background **0.74 (0.69-0.79)** p<0.001  Bangladeshi **0.43 (0.38-0.51)** p<0.001  Caribbean 0.98 (0.90-1.07)  Chinese 0.09 (0.72-1.06)  Gypsy/Romany **0.32 (0.19-0.53)** p<0.001  Indian **0.50 (0.45-0.55)** p<0.001  Irish 0.92 (0.77-1.10)  Pakistani **0.46 (0.42-0.50)** p<0.001  Traveller of Irish heritage **0.23 (0.10-0.56)** p<0.01  White and Asian 0.94 (0.83-1.08)  White and Black African 0.90 (0.75-1.09)  White and Black Caribbean **0.87 (0.78-0.96)** p<0.01  White British 1.00 | | | - |
| Pedersen  (2012) | Maricopa,  USA | Cross-sectional  (Surveillance program (ADDSP),  Population estimated using a  census,  Children age 8 (n=142,717),  1212 cases)  NOS: 6 | ASD ICD-9 | Ethnic minority status | *Number of cases/ total-number:*  In 2000  Hispanic 39/ 14,252  White 211/ 24,058  In 2002  Hispanic 53/ 15,348  White 195/ 25,252  In 2004  Hispanic 39/ 5,576  White 83/ 6,571  In 2006  Hispanic 138/ 17,577  White 295/ 19,664  *Prevalence (95% CI):* P< 0.005  In 2000  Hispanic **2.7 per 1000 (1.9-3.7)**  White 8.8 per 1000 (7.6-10.0)  In 2002  Hispanic **3.5 per 1000 (2.6-4.5)**  White 7.8 per 1000 (6.7-8.9)  In 2004  Hispanic **7.0 per 1000 (5.0-9.6)**  White 12.6 per 1000 (10.1-15.6)  In 2006  Hispanic **7.9 per 1000 (6.6-9.3)**  White 15.0 per 1000 (13.4-16.8) | | | - |
| Keen  (2010) | Lambeth and  Wandsworth,  UK | Retrospective case note  analysis  (Clinical service,  Population from the census,  137 cases in Lambeth and 258  cases in Wandsworth diagnosed between 1 September 1999 to 31 August 2005)  NOS: 4 | Autism-spectrum disorders ICD-10  (2/3 had childhood autism according to ICD-10) | Migration (descendants of migrants):  Mother´s birth region  Ethnic minority status:  Mother´s ethnicity  Both migration and ethnic minority status:  Mother´s ethnicity and whether mother is born in the UK or not | *Number of cases/ total-number:*  Migration:  UK  Other Europe  Africa  Caribbean  Asia  Ethnic minority status:  White  Black  Asian  Both migration and ethnic minority status:  White  UK-born  Immigrant  Black  UK-born  Immigrant  Asian  UK-born  Immigrant  *Rate ratio (95% CI):*  Migration:  UK  Other Europe  Africa  Caribbean  Asia  Ethnic minority status:  White  Black  Asian  *Adjusted rate ratio (95% CI):*  Both migration and ethnic minority status:  White  UK-born  Immigrant  Black  UK-born  Immigrant  Asian  UK-born  Immigrant | Lambeth:  48/ 44,015  8/ 5,581  56/ 6,482  14/ 1,283  10/ 2,310  27/ 41,332  97/ 17,932  13/ 3,219  19/ 30,973  8/ 10,359  25/ 10,581  69/ 7,351  2/ 1,680  10/ 1,539  1.00  1.31 (0.62-2.78)  **7.92 (5.39-11.6)**  **10.01 (5.53-18.1)**  **3.97 (2.01-7.84)**  1.00  **8.28 (5.41-12.7)**  **6.18 (3.19-12.0)**  1.00  1.26 (0.55-2.87)  **2.07 (1.14-3.75)**  **8.21 (4.94-13.63)**  1.01 (0.23-4.33)  **5.52 (2.57-11.83)** | Wandsworth:  152/ 48,858  20/ 5,276  47/ 4,621  13/ 470  22/ 3,402  163/ 55,404  78/ 6,908  16/ 4,416  132/ 41,888  31/ 13,516  19/ 4,384  57/ 2,524  0/ 1,994  16/ 2,422  1.00  1.22 (0.76-1.94)  **3.27 (2.36-4.53)**  **8.89 (5.08-15.5)**  **2.08 (1.33-3.25)**  1.00  **3.84 (2.93-5.02)**  1.23 (0.74-2.06)  1.00  0.73 (0.49-1.08)  0.74 (0.46-1.20)  **3.86 (2.84-5.26)**  - ( - )  1.13 (0.67-1.90) | Family size |
| Kogan  (2008) | USA | Cross-sectional  (Survey (NS-CSHCN),  Children with special health care needs, age <18, April 2005 to February 2007,  2088 cases)  NOS: 4 | Question “Does your child currently have autism or ASD?”  (All children with special health care needs) | Ethnic minority status | *Number of cases/ total-number (*Estimated frequency in population):*  Hispanic Any race -/ 64,000  Non-hispanic, White -/ 360,000  Non-hispanic, Black -/ 75,000  Non-hispanic, Other -/ 32,000  *Prevalence (%):*  Hispanic Any race 5.8  Non-hispanic, White 5.8  Non-hispanic, Black 4.9  Non-hispanic, Other 5.2  *Adjusted OR (95% CI):*  Hispanic Any race 0.97 (0.72-1.30)  Non-hispanic, White 1.00  Non-hispanic, Black **0.72 (0.57-0.91)**  Non-hispanic, Other 0.93 (0.71-1.23) | | | Child’s age, gender, Hispanic origin and race, primary language in home, household education level, poverty status, family structure, and region |
| Kamer  (2004) | Israel | Cross-sectional  (Register,  Children born 1983-1997  (n=1 236 000),  1004 cases)  NOS: 3 | Pervasive Developmental Disorders (PDD) including autism, Rett’s disorder, childhood disintegrative disorder, Asperger’s syndrome  and PDD-NOS | Migration (Both migrants and descendants of migrants):  Children´s birth country | *Number of cases/ total-number:*  Native Israelis of non-Ethiopian extraction 991/ 1,098,300  Native Israelis of Ethiopian extraction 13/ 15,600  Immigrants of non-Ethiopian extraction 59/ 110,300  Immigrants born in Ethiopia 0/ 11,800  *Prevalence:*  Native Israelis of non-Ethiopian extraction 9.0 per 10,000  Native Israelis of Ethiopian extraction 8.3 per 10,000  Immigrants of non-Ethiopian extraction 5.3 per 10,000  Immigrants born in Ethiopia 0.0 per 10,000  *OR (95% CI):*  Native Israelis of non-Ethiopian extraction **1.7 (1.3-2.2)**  Native Israelis of Ethiopian extraction 1.0  Native Israelis **1.85 (1.5–2.4)**  Immigrants 1.00 | | | - |
| Yeargin-Allsopp (2003) | Atlanta, USA | Cross-sectional  (Multiple-source surveillance (MADDSP) linked to birth certificate,  Population from census,  Children age 3-10 in 1996 (n= 289 456),  987 cases)  NOS: 5 | Autistic Disorder, PDD-NOS, and Asperger Disorder DSM-IV  (64% had MR) | Ethnic minority status | *Number of cases:*  Male  White 457  Black 305  Other 25  Female  White 120  Black 70  Other 7  Total  White 577  Black 375  Other 32  *Prevalence (95% CI):*  Male  White 5.3 per 1000 (4.8-5.8)  Black 5.4 per 1000 (4.8-6.1)  Other 4.3 per 1000 (2.8-6.4)  Female  White 1.5 per 1000 (1.2-1.8)  Black 1.3 per 1000 (1.0-1.6)  Other 1.4 per 1000 (0.5-2.8)  Total  White 3.4 per 1000 (3.2-3.7)  Black 3.4 per 1000 (3.0-3.7)  Other 2.9 per 1000 (2.0-4.1) | | | - |
| Croen  (2002) | California,  USA | Retrospective cohort  (Regional centers,  Linked to the birth register, Children born 1987-1994  (n= 3,497,870),  4356 cases)  NOS: 8 | Full syndrome autism,  defined by the California Department of Developmental Services.  (36% had mental  retardation) | Migration  (descendants of migrants):  Maternal birthplace  Ethnic minority status:  Maternal race/ethnicity | Migration:  *-Number of cases/ total-number:*  California 1,880/ 1,363,197  Other United States 974/ 636,340  Mexico 507/ 888,534  Other 995/ 609,799  *-Rate ratio (95% CI):*  California 1.0  Other United States 1.1 (1.0-1.2)  Mexico **0.4 (0.4-0.5)**  Other **1.2 (1.1-1.3)**  *-Adjusted rate ratio (95% CI):*  California 1.0  Other United States 0.9 (0.8-1.0)  Mexico **0.6 (0.5-0.7)**  Other 1.1 (1.0-1.2)  Ethnic minority status:  *-Number of cases/ total-number:*  White 2,059/ 1,385,715  Hispanic 1,213/ 1,483,508  Black 499/ 275,637  Asian 337/ 200,198  Other 248/ 152,812  *-Rate ratio (95% CI):*  White 1.0  Hispanic 0.6 (0.5-0-6)  Black **1.2 (1.1-1.3)**  Asian 1.1 (1.0-1.3)  Other 1.1 (1.0-1.2)  *-Adjusted rate ratio (95% CI):*  White 1.0  Hispanic 1.1 (1.0-1.3)  Black **1.6 (1.5-1.8)**  Asian 1.0 (0.9-1.1)  Other 1.0 (0.9-1.2) | | | Child sex, birth weight, plurality, birth order, maternal age, maternal ethnicity and maternal education  Child sex, birth weight, plurality, birth order, maternal age, maternal birth place and maternal education |
| Wing  (1980) | Camberwell,  UK | Cross-sectional  (Survey,  Children aged <15,  17 cases)  NOS: 3 | Autism (nuclear and non nuclear) | Migration (Generation not described):  Father´s birth region | *Number of cases*:  British and European 12  New commonwealth 5  *Prevalence:* Too small for the calculation of the significance  British and European 4.4 per 10,000  New commonwealth 6.3 per 10,000 | | | - |
| NOS = Newcastle-Ottawa Scale; ASD = autism spectrum disorder; PDD-NOS = pervasive developmental disorder-not otherwise specified; DSM = Diagnostic and Statistical Manual of Mental Disorders; ICD = International Classification of Diseases; OR= odds ratio; CI = confidence interval | | | | | | | | |

| **Table S5. ASD–ID** | | | | | | | |
| --- | --- | --- | --- | --- | --- | --- | --- |
| **First**  **Author**  (publica-tion data) | **Location** | **Design**  (Source of data, Population, Sample size and description)  **Study quality with NOS** | **Case**  **ascertainment** | **Exposure** (migration: either migrants or descendants of migrants) | **Results** | | **Adjustment factor** |
| Fairthorne (2017) | Western Australia | Retrospective cohort (Register,  All women with a live-born child in Western Australia born 1994-2005 (n = 134 204),  347 cases diagnosed before 2011)  NOS: 7 | ASD without ID DSM-IV and DSM-IV-TR  (An IQ test was performed in all cases unless the child obviously did not have ID or the child was unable to be assessed) | Migration (descendants of migrants): Maternal immigration status depending on birthplace | *Number of cases/ total-number:*  Non-immigrant 262/ 97,731  Immigrant 85/ 36,473  *Adjusted OR (95% CI):*  Non-immigrant 1.0  Immigrant **0.62 (0.5-0.8)** | | Demographic factors |
| Hewitt  (2016) | Minnea-polis,  USA | Cross-sectional  (Multiple source (MSASDPP),  Compared to population estimated  using a census,  Children ages 7-9 (n=12,329),  124 cases)  NOS: 4 | Autistic disorder,  PDD-NOS  (including atypical  autism), and  Asperger disorder  DSM-IV-TR, without Intellectual disability (IQ <70) | Ethnic minority status | *Number and percent of ASD without ID:* p< 0.05  White 71 of 89 ASD cases (80%)  Black (non-Somali) 30 of 43 ASD cases (70%)  Somali 0 of 20 ASD cases (0%)  Hispanic 14 of 18 ASD cases (78%)  *Prevalence of ASD:*  White 27.7 per 1000  Black (non-Somali) 16.0 per 1000  Somali 30.8 per 1000  Hispanic 12.5 per 1000  *Prevalence of ASD without ID (calculated):*  White 22.2 per 1000  Black (non-Somali) **11.2 per 1000**  Somali **0.0 per 1000**  Hispanic **9.8 per 1000** | | **-** |
| Lehti (2015) | Finland | Nested case-control  (Register,  Children born  1987-2005,  1 783 cases diagnosed by 2007,  4 controls/case matched by date of birth (±30 days), region of birth, sex, and residence in Finland)  NOS: 8 | Asperger’s syndrome  ICD-10: F84.5 | Migration  (descendants of migrants):  -Primary  analysis:  Parental immigration  status  -Secondary  analysis:  Maternal and paternal region of birth | Primary analysis:  *Number of cases/ controls:*  Both parents Finnish 1,719/ 6,752  Mother only immigrated 23/ 88  Father only immigrated 35/ 133  Both parents immigrated 6/ 133  *OR (95% CI):*  Both parents Finnish 1.0  Mother only immigrated 1.0 (0.6-1.6)  Father only immigrated 1.0 (0.7-1.5)  Both parents immigrated **0.2 (0.1-0.4)**  Secondary analysis:  *Number of cases/ controls:*  Mothers  Finnish 1,752/ 6,883  Western countries 8 /37  Former Soviet Union+Yugoslavia 8/ 55  Sub-Saharan Africa 1/ 71  North Africa, Middle East 1/ 18  Asia 8/ 35  Fathers  Finnish 1,739/ 6,836  Western countries 20/ 71  Former Soviet Union+Yugoslavia 4/ 27  Sub-Saharan Africa 3/ 91  North Africa, Middle East 8/ 47  Asia 2 /25  *OR (95% CI):*  Mothers  Finnish 1.0  Western countries 0.9 (0.4-1.8)  Former Soviet Union+Yugoslavia 0.6 (0.3-1.2)  Sub-Saharan Africa **0.1 (0.01-0.4)**  North Africa, Middle East 0.2 (0.03-1.6)  Asia 0.9 (0.4-1.9)  Fathers  Finnish 1.0  Western countries 1.1 (0.7-1.8)  Former Soviet Union+Yugoslavia 0.6 (0.2-1.6)  Sub-Saharan Africa **0.1 (0.04-0.4)**  North Africa, Middle East 0.7 (0.3-1.4)  Asia 0.3 (0.1-1.3) | Primary analysis:  *Adjusted OR (95% CI):*  Both parents Finnish 1.0  Mother only immigrated 1.1 (0.7-1.7)  Father only immigrated 1.0 (0.7-1.4)  Both parents immigrated **0.2 (0.1–0.4)**  Secondary analysis:  *Adjusted OR (95% CI):*  Mothers  Finnish 1.0  Western countries 0.9 (0.4-2.0)  Former Soviet Union+Yugoslavia 0.6 (0.3-1.3)  Sub-Saharan Africa **0.1 (0.01-0.5)**  North Africa, Middle East 0.3 (0.03-1.9)  Asia 0.9 (0.4-2.0)  Fathers  Finnish 1.0  Western countries 1.0 (0.6-1.7)  Former Soviet Union+Yugoslavia 0.6 (0.2-1.6)  Sub-Saharan Africa **0.2 (0.05-0.5)**  North Africa, Middle East 0.7 (0.3-1.4)  Asia 0.3 (0.1-1.3) | Maternal age,  maternal smoking  and parity  Maternal age,  maternal smoking  and parity |
| Kim Van Naarden (2015) | Atlanta,  USA | Cross-sectional  (Multiple-source surveillance (MADDSP),  Population estimated using a census,  Children aged 8,  752 cases)  NOS: 4 | ASD DSM-IV  without a comorbid Intellectual disability (defined as IQ<70) | Ethnic minority status | *Proportions of cases in 2010:*  White non-Hispanic Males  IQ>85 55.9% of 246 ASD cases  IQ 71-85 19.1% of 246 ASD cases  Black non-Hispanic Males  IQ>85 29.6% of 227 ASD cases  IQ 71-85 24.0% of 227 ASD cases  White non-Hispanic Females  IQ>70 64.7% of 41 ASD cases  Black non-Hispanic Females  IQ>70 54.2% of 64 ASD cases  *Prevalence in 2010:*  White non-Hispanic Males  IQ>85 15.3 per 1000  IQ 71-85 5.2 per 1000  Black non-Hispanic Males  IQ>85 5.5 per 1000  IQ 71-85 4.5 per 1000  White non-Hispanic Females  IQ>70 2.8 per 1000  Black non-Hispanic Females  IQ>70 3.1 per 1000 | | **-** |
| Van der Ven  (2013) | Nether-lands | Retrospective cohort  (Register,  Children born 1998-2007 (n = 106 953),  368 cases)  NOS: 8 | Asperger syndrome  (DSM-IV code 299.80) or PDD- NOS (299.80) | Migration  (descendants of migrants):  Maternal  country of birth  Paternal immigrant status | *Number of cases/ total-number:*  Netherlands 320/ 80,354  Developing countries 32/ 19,948  Developed countries 16/ 6,651 | *Adjusted rate ratio (95% CI):*  Netherlands 1.0  Developing countries  All **0.4 (0.3-0.6)**  Turkey 0.5 (0.2-1.2)  Morocco **0.2 (0.1-0.4)**  Suriname and Dutch Antilles 1.0 (0.5-1.9)  Other **0.3 (0.2-0.7)**  Developed countries  All 0.6 (0.4-1.1)  *Rate ratio (95% CI):*  Native Dutch 1.0  A Dutch mother and a foreign father 0.8 (0.5-1.3) | Gender and  paternal age |
| Magnusson  (2012) | Stockholm,  Sweden | Nested case-control  (Register,  Children aged 0-17 living in Stockholm 2001-2007 (n = 589  114),  2269 cases,  10 controls/case matched by birth date and gender)  NOS: 8 | Autism diagnosis without a comorbid Intellectual disability  (defined as IQ<70) recorded in Swedish Healthcare registers | Migration  (Both migrants and descendants of migrants):  Parental  migration status and maternal country of birth by geographical  sub region | *Number of cases/ controls:*  Both parents born in Sweden 1,963/18,116  Both parents born abroad 306/ 4,925  Maternal country of birth:  Northern Africa 8/ 175  Eastern Africa 26/ 521  Other African 2/ 82  Northern America 1/ 20  Latin America/Caribbean 35/ 456  Southern Asia 26/ 556  Western Asia 64/ 1,627  Other Asian 13/ 160  Northern Europe 74/ 572  Eastern Europe 35/ 308  Southern Europe 14/ 355  Western Europe 8/ 85  *OR (95% CI):*  Both parents born in Sweden 1.0  Both parents born abroad **0.5 (0.5-0.6)**  Maternal country of birth:  Northern Africa **0.4 (0.2-0.8)**  Eastern Africa **0.4 (0.3-0.6)**  Other African **0.2 (0.1-0.6)**  Northern America not applicable  Latin America/Caribbean **0.7 (0.5-0.9)**  Southern Asia **0.5 (0.3-0.6)**  Western Asia **0.4 (0.3-0.5)**  Other Asian 0.6 (0.4-1.1)  Northern Europe 1.2 (0.9-1.5)  Eastern Europe 1.0 (0.7-1.3)  Southern Europe **0.3 (0.2-0.6)**  Western Europe 0.9 (0.5-1.8)  Time since mother’s immigration to Sweden in relation to the child’s birth  *Number of cases/ controls:*  >15 years before birth 30/ 457  10-14 years before birth 39/ 579  5-9 years before birth 74/ 970  1-4 years before birth 82/ 1,284  Migrated in the year before birth 33/ 454  Migrated within 1 year after birth 6/ 138  1-4 years after birth 23/ 437  >5 years after birth 9/ 514 | *Adjusted OR (95% CI):*  Both parents born in Sweden 1.0  Both parents born abroad **0.5 (0.4-0.6)**  Maternal country of birth:  Northern Africa **0.3 (0.2-0.7)**  Eastern Africa **0.3 (0.2-0.5)**  Other African **0.2 (0.0-0.7)**  Northern America not applicable  Latin America/Caribbean **0.5 (0.4-0.8)**  Southern Asia **0.3 (0.2-0.5)**  Western Asia **0.3 (0.2-0.4)**  Other Asian 0.6 (0.4-1.1)  Northern Europe 1.1 (0.8-1.4)  Eastern Europe 0.8 (0.6-1.2)  Southern Europe **0.3 (0.2-0.5)**  Western Europe 0.8 (0.4-1.7)  Time since mother’s immigration to Sweden in relation to the child’s birth  *Adjusted OR (95% CI):*  >15 years before birth 1.0  10-14 years before birth 1.1 (0.6-1.9)  5-9 years before birth 1.5 (0.9-2.5)  1-4 years before birth 1.5 (0.9-2.4)  Migrated in the year before birth 1.7 (0.9-3.0)  Migrated within 1 year after birth 1.0 (0.4-2.6)  1-4 years after birth 1.3 (0.7-2.4)  >5 years after birth 0.5 (0.2-1.2) | Maternal and  paternal age at child’s birth and family disposable income at child’s birth or in early life  Mother’s region of birth and time since immigration in relation to child birth mutually adjusted |
| Haglund  (2011) | Malmoe,  Sweden | Nested case-control  (Clinical record,  Linked to the birth  register,  Children born 1980-2005 (N = 68,964),  93 cases,  Controls matched by birth place and year of birth)  NOS: 7 | Asperger syndrome DSM-IV or ICD-10 | Migration  (descendants of migrants):  Maternal  country of birth | *Number of cases:*  Mother born outside Sweden 12  *OR (95% CI):*  Mother born outside Sweden **0.5 (0.3–0.8)** | *Adjusted OR (95% CI):*  Mother born outside  the Nordic countries **0.6 (0.3-0.97)** | Maternal age ≥40 years, parity, maternal smoking,  gender, birth <37 weeks, and gestational age-adjusted weight Standard Deviation (SD) scores (continuous) |
| ASD = autism spectrum disorder; ID = intellectual disability; NOS = Newcastle-Ottawa Scale; PDD-NOS = pervasive developmental disorder-not otherwise specified; DSM = Diagnostic and Statistical Manual of Mental Disorders; ICD = International Classification of Diseases; OR= odds ratio; CI = confidence interval | | | | | | | |

| **Table S6. ASD+ID** | | | | | | | | |
| --- | --- | --- | --- | --- | --- | --- | --- | --- |
| **First**  **Author**  (publica-tion data) | **Location** | **Design**  (Source of data, Population, Sample size and description)  **Study quality with NOS** | **Case ascertainment** | **Exposure** (migration: either migrants or descendants of migrants) | **Results** | | | **Adjustment factor** |
| Fairthorne (2017) | Western Australia | Retrospective cohort (Register,  All women with a live-born child in Western Australia born 1994-2005 (n = 134 204),  1028 cases diagnosed before 2011)  NOS: 7 | ASD with ID DSM-IV and DSM-IV-TR  (An IQ test was performed in all cases unless the child obviously did not have ID or the child was unable to be assessed) | Ethnic minority status:  Maternal ethnicity  Migration (descendants of migrants): Maternal immigration status depending on birthplace  Both migration and ethnic minority status: | Ethnic minority status:  *Number of cases/ total-number:*  Caucasian 937/ 119,801  Asian 56/ 6,578  Black 7/ 601  Indigenous* 23/ 5,393  *Australian Aboriginal or Torres Strait Islander  *OR (95% CI):*  Caucasian 1.0  Asian 1.09 (0.8-1.4)  Black 1.48 (0.7-3.1)  Indigenous **0.54 (0.4-0.8)**  Migration:  *Number of cases/ total-number:*  Non-immigrant 752/ 97,731  Immigrant 276/ 36,473  *OR (95% CI):*  -  Both migration and ethnic minority status:  *Number of cases/ total-number:*  Caucasian nonimmigrant 212/ 27,857  Asian immigrant from Central Asia 18/ 1,782  Asian immigrant from South Asia 30/ 2,777  Asian immigrant from Other Asia 6/ 1,730  Black immigrant from East Africa 7/ 253  Indigenous 0/ 330  *OR (95% CI):*  Caucasian nonimmigrant 1.0  Asian immigrant from Central Asia 1.28 (0.8-2.0)  Asian immigrant from South Asia 1.37 (0.9-2.0) Asian immigrant from Other Asia **0.44(0.2-0.98)**  Black immigrant from East Africa **3.57 (1.7-7.6)**  Indigenous **0.54 (0.4-0.8)** | | Ethnic minority status:  *Adjusted OR (95% CI):*  Caucasian 1.0  Asian 0.77 (0.6-1.02)  Black -  Indigenous **0.52 (0.3-0.8)**  Migration:  *Adjusted OR (95% CI):*  Non-immigrant 1.0  Immigrant **0.64 (0.6-0.7)**  Both migration and ethnic minority status:  *Adjusted OR (95% CI):*  Caucasian nonimmigrant 1.0  Asian immigrant from Central Asia 0.83 (0.5-1.3)  Asian immigrant from South Asia 0.83 (0.6-1.2)  Asian immigrant from Other Asia -  Black immigrant from East Africa -  Indigenous **0.51 (0.3-0.8)** | Maternal age, parity, socioeconomic status, and birth-year group  Demographic factors  Maternal age, parity, socioeconomic status, and birth-year group |
| Hewitt  (2016) | Minnea-polis,  USA | Cross-sectional  (Multiple source (MSASDPP), Compared to  population estimated using a census,  Children ages 7-9 (n=12,329),  60 cases)  NOS: 4 | Autistic disorder, PDD-NOS (including  atypical autism), and Asperger disorder DSM-IV-TR, with Intellectual disability IQ<70 | Ethnic minority status | *Number and percent of ASD with ID:* p< 0.05  White 18 of 89 ASD cases (20%)  Black (non-Somali) 13 of 43 ASD cases (30%)  Somali 20 of 20 ASD cases (100%)  Hispanic 4 of 18 ASD cases (22%)  *Prevalence of ASD:*  White 27.7 per 1000  Black (non-Somali) 16.0 per 1000  Somali 30.8 per 1000  Hispanic 12.5 per 1000  *Prevalence of ASD with ID (calculated):*  White 5.5 per 1000  Black (non-Somali) 4.8 per 1000  Somali **30.8 per 1000**  Hispanic **2.8 per 1000** | | | - |
| Kim Van Naarden (2015) | Atlanta,  USA | Cross-sectional  (Multiple-source surveillance (MADDSP),  Population estimated using a census,  Children aged 8,  752 cases)  NOS: 4 | ASD DSM-IV with a comorbid intellectual disability (defined as IQ<70) | Ethnic minority status | *Proportions of cases in 2010:*  White non-Hispanic Males 25.0% of 246 ASD cases  Black non-Hispanic Males 46.4% of 227 ASD cases  White non-Hispanic Females 35.3% of 41 ASD cases  Black non-Hispanic Females 45.8% of 64 ASD cases  *Prevalence in 2010:*  White non-Hispanic Males 6.8 per 1000  Black non-Hispanic Males 8.7 per 1000  White non-Hispanic Females 1.5 per 1000  Black non-Hispanic Females 2.6 per 1000 | | | - |
| Becerra  (2014) | Los Angeles,  USA | Retrospective cohort  (Regional centers,  Linked to the birth register,  Children born 1995-2006 (n=1 626 354),  7540 cases with autistic disorder, 806 of which with comorbid mental retardation, diagnosed at age 3-5 during 1998-2009)  NOS: 7 | Autistic disorder DSM-IV (ICD-9-CM code: 299.00) | Migration  (descendants of migrants):  Maternal  nativity  Both migration  and ethnic minority status:  Maternal  race/ethnicity  and whether  mother is US-  or foreign-born | Migration:  *Number of cases/ total-number:*  US-born white 1477/ 236,347  Mexico 1792/ 548,977  Central/South America 760/ 157,147  China 145/ 29,666  Japan 29/ 5,815  Korea 136/ 22,206  Philippines 266/ 35,306  Vietnam 179/ 19,287  *RR (95% CI):*  US-born white 1.00  Mexico **0.52 (0.49-0.56)**  Central/South America **0.77 (0.71-0.84)**  China **0.78 (0.66-0.93)**  Japan 0.82 (0.57-1.18)  Korea 0.98 (0.82-1.16)  Philippines **1.21 (1.06-1.37)**  Vietnam **1.48 (1.27-1.74)**  Both migration and ethnic minority status:  *Number of cases/ total-number:*  White  US-born 1477/ 236,347  Foreign-born 420/ 63,464  Black  US-born 526/ 123,316  Foreign-born 92/ 10,093  Hispanic  US-born 1376/ 316,565  Foreign-born 2594/ 711,825  Asian  US-born 138/ 21,678  Foreign-born 917/ 143,066  *Number of cases/ total-number:*  White  US-born 1.00  Foreign-born 1.06 (0.95-1.18)  Black  US-born **0.68 (0.62-0.75)**  Foreign-born **1.46 (1.18-1.80)**  Hispanic  US-born **0.70 (0.65-0.75)**  Foreign-born **0.58 (0.55-0.62)**  Asian  US-born 1.02 (0.85-1.21)  Foreign-born 1.03 (0.94-1.11) | Migration:  *Adjusted RR (95% CI):*  US-born white 1.00  Mexico **0.76 (0.70-0.82)**  Central/South America 1.08 (0.98-1.19)  China **0.66 (0.56-0.79)**  Japan 0.71 (0.49-1.02)  Korea 0.92 (0.77-1.10)  Philippines **1.23 (1.08-1.40)**  Vietnam **1.45 (1.24-1.70)**  *Additionally adjusted RR (95% CI):*  US-born white 1.00  Mexico 0.95 (0.86-1.05)  Central/South America **1.25 (1.13-1.39)**  China **0.74 (0.62-0.89)**  Japan 0.72 (0.50-1.04)  Korea 0.95 (0.80-1.14)  Philippines **1.23 (1.08-1.41)**  Vietnam **1.58 (1.35-1.86)**  Both migration and ethnic minority status:  *Adjusted RR (95% CI):*  White  US-born 1.00  Foreign-born 1.02 (0.91-1.14)  Black  US-born 1.00 (0.90-1.10)  Foreign-born **1.59 (1.28-1.96)**  Hispanic  US-born 1.08 (1.00-1.17)  Foreign-born **0.85 (0.79-0.91)**  Asian  US-born 1.03 (0.86-1.22)  Foreign-born 0.98 (0.90-1.06)  *Additionally adjusted RR (95% CI):*  White  US-born 1.00  Foreign-born 1.05 (0.94-1.17)  Black  US-born 1.04 (0.94-1.15)  Foreign-born **1.65 (1.33-2.05)**  Hispanic  US-born **1.15 (1.06-1.24)**  Foreign-born 1.05 (0.97-1.14)  Asian  US-born 1.02 (0.85-1.21)  Foreign-born 1.04 (0.95-1.13) | | Maternal age, type of birth, parity, infant gender, year of birth, gestational age, birth weight, trimester start of prenatal care, and any pregnancy  complication  Additionally  adjusted for  maternal education and insurance type  Maternal age, type of birth, parity, infant gender, year of birth, gestational age, birth weight,  trimester start of  prenatal care, and any pregnancy  complication  Additionally  adjusted for  maternal education and insurance type |
|  |  |  | Autistic  disorder DSM-IV (ICD-9-CM code: 299.00) and Mental  retardation  DSM-IV (ICD-9-CM codes: 317, mild; 318.0, moderate;  318.1, severe;  318.2, profound; 319, MR unspecified) | Migration  (descendants of migrants):  Maternal  nativity  Both migration  and ethnic minority status:  Maternal  race/ethnicity  and whether  mother is US-  or foreign-born | Migration:  *Number of cases/ total-number:*  US-born white 122/ 236,347  Mexico 190/ 548,977  Central/South America 86/ 157,147  China 19/ 29,666  Japan 2/ 5,815  Korea 6/ 22,206  Philippines 35/ 35,306  Vietnam 20/ 19,287  *RR (95% CI):*  US-born white 1.00  Mexico **0.67 (0.53-0.84)**  Central/South America 1.06 (0.80-1.40)  China 1.24 (0.76-2.01)  Japan 0.67 (0.16-2.69)  Korea 0.52 (0.23-1.19)  Philippines **1.92 (1.32-2.80)**  Vietnam **2.01 (1.25-3.22)**  Both migration and ethnic minority status:  *Number of cases/ total-number:*  White  US-born 122/ 236,347 Foreign-born 35/ 63,464  Black  US-born 78/ 123,316  Foreign-born 13/ 10,093  Hispanic  US-born 161/ 316,565  Foreign-born 281/ 711,825  Asian  US-born 14/ 21,678  Foreign-born 102/ 143,066  *RR (95% CI):*  White  US-born 1.00  Foreign-born 1.07 (0.73-1.56)  Black  US-born 1.23 (0.92-1.63)  Foreign-born **2.49 (1.41-4.42)**  Hispanic  US-born 0.99 (0.78-1.25)  Foreign-born **0.76 (0.62-0.95)**  Asian  US-born 1.25 (0.72-2.18)  Foreign-born **1.38 (1.06-1.80)** | Migration:  *Adjusted RR (95% CI):*  US-born white 1.00  Mexico 0.79 (0.62-1.01)  Central/South America 1.20 (0.90-1.60)  China 1.19 (0.73-1.93)  Japan not applicable  Korea not applicable  Philippines **1.92 (1.31-2.80)**  Vietnam **1.96 (1.22-3.16)**  *Additionally adjusted RR (95% CI):*  US-born white 1.00  Mexico 0.94 (0.70-1.26)  Central/South America **1.42 (1.03-1.96)**  China 1.25 (0.76-2.05)  Japan not applicable  Korea not applicable  Philippines **1.98 (1.36-2.90)**  Vietnam **2.07 (1.28-3.35)**  Both migration and ethnic minority status:  *Adjusted RR (95% CI):*  White  US-born 1.00  Foreign-born 1.06 (0.73-1.55)  Black  US-born **1.42 (1.06-1.90)**  Foreign-born **2.56 (1.44-4.53)**  Hispanic  US-born **1.30 (1.02-1.66)**  Foreign-born 0.91 (0.73-1.14)  Asian  US-born 1.31 (0.75-2.27)  Foreign-born **1.36 (1.04-1.77)**  *Additionally adjusted RR (95% CI):*  White  US-born 1.00  Foreign-born 1.08 (0.74-1.57)  Black  US-born **1.47 (1.09-1.97)**  Foreign-born **2.67 (1.50-4.74)**  Hispanic  US-born **1.35 (1.04-1.73)**  Foreign-born 1.01 (0.79-1.30)  Asian  US-born 1.30 (0.75-2.27)  Foreign-born **1.41 (1.08-1.84)** | | Maternal age, type of birth, parity, infant gender, year of birth, gestational age, birth weight,  trimester start of  prenatal care, and any pregnancy complication  Additionally  adjusted for  maternal education and insurance type  Maternal age, type of birth, parity, infant gender, year of birth, gestational  age, birth weight, trimester start of  prenatal care, and any pregnancy  complication  Additionally  adjusted for  maternal education and insurance type |
| Lehti  (2013) | Finland | Nested case-control (Register,  Children born 1987-2005,  1132 cases diagnosed by 2007,  4 controls/case matched by date of birth (±30 days), region of birth, sex, and residence in  Finland)  NOS: 9 | Childhood autism (ICD-9: code 299.0 and  ICD-10: F84.0) | Migration  (descendants of migrants):  -Primary  analysis:  Parental immigration status  -Secondary  analysis:  Maternal and paternal region and country of birth | Primary analysis:  *Number of cases/ controls:*  Both parents Finnish 1,035/ 4,265  Mother only immigrated 35 /80  Father only immigrated 23/ 77  Both parents immigrated 39/ 93  *OR (95% CI):*  Both parents Finnish 1.0  Mother only immigrated **1.8 (1.2-2.7)**  Father only immigrated 1.2 (0.8-2.0)  Both parents immigrated **1.8 (1.2-2.6)**  Secondary analysis (region):  *Number of cases/ controls:*  Mothers  Finnish 1,058/ 4,340  Western countries 10/ 29  Former Soviet Union+Yugoslavia 29/ 65  Sub-Saharan Africa 10/ 36  North Africa, Middle East 2/ 14  Asia 18/ 28  Fathers  Finnish 1,069/ 4,344  Western countries 8/ 43  Former Soviet Union+Yugoslavia 15/ 35  Sub-Saharan Africa 13/ 38  North Africa, Middle East 9/ 38  Asia 13/ 12  *OR (95% CI):*  Mothers  Finnish 1.0  Western countries 1.4 (0.7-2.9)  Former Soviet Union+Yugoslavia **1.8 (1.2-2.8)**  Sub-Saharan Africa 1.2 (0.6-2.4)  North Africa, Middle East 0.6 (0.1-2.6)  Asia **2.6 (1.4-4.7)**  Fathers  Finnish 1.0  Western countries 0.7 (0.4-1.6)  Former Soviet Union+Yugoslavia1.7(0.95-3.2)  Sub-Saharan Africa 1.4 (0.7-2.8)  North Africa, Middle East 1.0 (0.5-2.0)  Asia **4.4 (2.0-9.6)**  Secondary analysis (country):  *Number of cases/ controls:*  Mothers  Finnish 1,058/ 4,340  Former Yugoslavia 6/ 8  Former Soviet Union 22/ 50  Somalia 6/ 34  Thailand 3/ 13  Vietnam 5/ 9  Fathers  Finnish 1,069/ 4,344  Former Yugoslavia 8/ 8  Former Soviet Union 5/ 21  Somalia 8/ 31  Turkey 1/ 10  Vietnam 8/ 5  *OR (95% CI):*  Mothers  Finnish 1.0  Former Yugoslavia **3.0 (1.1-8.8)**  Former Soviet Union **1.7 (1.05-2.9)**  Somalia 0.7 (0.3-1.8)  Thailand 1.0 (0.3-3.6)  Vietnam **7.0 (2.3-20.9)**  Fathers  Finnish 1.0  Former Yugoslavia **4.0 (1.5-10.6)**  Former Soviet Union 0.9 (0.4-2.5)  Somalia 1.1 (0.5-2.6)  Turkey 0.4 (0.1-3.3)  Vietnam **6.4 (2.1-19.7)** | Primary analysis:  *Adjusted OR (95% CI):*  Both parents Finnish 1.0  Mother only immigrated **1.8 (1.2-2.7)**  Father only immigrated 1.3 (0.8-2.1)  Both parents immigrated **1.8 (1.2-2.7)**  Secondary analysis (region):  *Adjusted OR (95% CI):*  Mothers  Finnish 1.0  Western countries 1.4 (0.7-2.9)  Former Soviet Union+Yugoslavia **1.8 (1.2-2.9)**  Sub-Saharan Africa 1.2 (0.6-2.6)  North Africa, Middle East 0.6 (0.1-2.7)  Asia **2.6 (1.4-4.7)**  Fathers  Finnish 1.0  Western countries 0.7 (0.4-1.6)  Former Soviet Union+Yugoslavia 1.8 (0.97-3.3)  Sub-Saharan Africa 1.5 (0.8-3.0)  North Africa, Middle East 1.0 (0.5-2.1)  Asia **4.4 (2.0-9.5)**  Secondary analysis (country):  *Adjusted OR (95% CI):*  Mothers  Finnish 1.0  Former Yugoslavia **3.2 (1.1-9.1)**  Former Soviet Union **1.7 (1.05-2.9)**  Somalia 0.8 (0.3-1.9)  Thailand 1.0 (0.3-3.7)  Vietnam **7.0 (2.3-21.2)**  Fathers  Finnish 1.0  Former Yugoslavia **4.1 (1.5-10.9)**  Former Soviet Union 0.9 (0.4-2.5)  Somalia 1.2 (0.5-2.8)  Turkey 0.5 (0.1-3.5)  Vietnam **6.4 (2.1-19.5)** | | Parental age  Parental age  Parental age |
| Van der  Ven  (2013) | Netherlands | Retrospective cohort  (Register,  Children born 1998-2007 (n = 106 953),  150 cases)  NOS: 8 | Autistic disorder (DSM-IV code 299.00) | Migration  (descendants of migrants):  Maternal  country of birth  Paternal migrant status | *Number of cases/ total-number:*  Netherlands 100/ 80,354  Developing countries 37/ 19,948  Developed countries 13/ 6,651 | *Adjusted rate ratio (95% CI):*  Netherlands 1.0  Developing countries  All 1.4 (0.9-2.4)  Turkey 1.8 (0.6-4.9)  Morocco 0.8 (0.3-1.9)  Suriname and Dutch Antilles 1.8 (0.6-5.4)  Other **2.3 (1.1-4.8)**  Developed countries  All 1.6 (0.8-3.5)  *Rate ratio (95% CI):*  Native Dutch 1.0  A Dutch mother and a foreign father **2.6 (1.4-5.0)** | | Gender and  paternal age |
| Magnus-son  (2012) | Stockholm,  Sweden | Nested case-control  (Register,  Children aged 0-17 living in Stockholm  2001-2007 (n = 589 114),  1649 cases,  10 controls/case matched by birth date and gender)  NOS: 8 | Autism diagnosis with a comorbid  Intellectual disability (IQ <  70) recorded in  Swedish Healthcare registers | Migration (Both migrants and descendants of migrants):  Parental  migration status and maternal  country of birth by geographical  sub region | *Number of cases/ controls:*  Both parents born in Sweden 1,159/13,357  Both parents born abroad 490/ 3,646  Maternal country of birth:  Northern Africa 23/ 123  Eastern Africa 96/ 414  Other African 28/ 66  Northern America 0/ 11  Latin America/Caribbean 59/334  Southern Asia 59/ 415  Western Asia 117/ 1,165  Other Asian 13/ 117  Northern Europe 40/ 445  Eastern Europe 22/ 232  Southern Europe 27/ 251  Western Europe 6/ 66  *OR (95% CI):*  Both parents born in Sweden 1.0  Both parents born abroad **1.5 (1.3-1.7)**  Maternal country of birth:  Northern Africa **2.0 (1.3-3.1)**  Eastern Africa **2.5 (2.0-3.1)**  Other African **3.6 (2.4-5.3)**  Northern America not applicable  Latin America/Caribbean **1.9 (1.4-2.4)**  Southern Asia **1.6 (1.2-2.1)**  Western Asia 1.1 (0.9-1.3)  Other Asian 1.1 (0.7-1.9)  Northern Europe 1.1 (0.8-1.6)  Eastern Europe 1.0 (0.7-1.5)  Southern Europe 1.3 (0.9-1.8)  Western Europe 1.1 (0.5-2.3)  Time since mother’s immigration to Sweden in relation to the child’s birth  *Number of cases/ controls:*  >15 years before birth 38/ 356  10-14 years before birth 51/ 413  5-9 years before birth 108/ 733  1-4 years before birth 149/ 966  Migrated in the year before birth 65/ 333  Migrated within 1 year after birth 19/ 102  1-4 years after birth 34/ 334  >5 years after birth 19/ 344 | *Adjusted OR (95% CI):*  Both parents born in Sweden 1.0  Both parents born abroad 1.2 (1.0-1.4)  Maternal country of birth:  Northern Africa 1.5 (0.9-2.4)  Eastern Africa **1.9 (1.5-2.5)**  Other African **3.5 (2.5-5.6)**  Northern America not applicable  Latin America/Caribbean **1.6 (1.2-2.2)**  Southern Asia 1.3 (0.9-1.7)  Western Asia 0.9 (0.7-1.2)  Other Asian 1.1 (0.6-2.0)  Northern Europe 0.9 (0.6-1.2)  Eastern Europe 0.8 (0.5-1.3)  Southern Europe 1.1 (0.8-1.7)  Western Europe 1.0 (0.4-2.2)  Time since mother’s immigration to Sweden in relation to the child’s birth  *Adjusted OR (95% CI):*  >15 years before birth 1.0  10-14 years before birth 1.2 (0.7-1.9)  5-9 years before birth 1.2 (0.8-1.8)  1-4 years before birth 1.4 (0.9-2.1)  Migrated in the year before birth **1.8 (1.1-2.9)**  Migrated within 1 year after birth 1.2 (0.6-2.4)  1-4 years after birth 0.9 (0.5-1.6)  >5 years after birth 0.6 (0.3-1.2) | | Maternal and  paternal age at  child’s birth and  family disposable income at child’s birth or in early life  Mother’s region of birth and time since immigration in  relation to child  birth mutually  adjusted |
| Pedersen  (2012) | Maricopa,  USA | Cross-sectional  (Surveillance program (ADDSP),  Population estimated using a census,  Children age 8 (n=142,717),  283 cases)  NOS: 6 | ASD ICD-9 with ID according to clinician review | Ethnic minority status | *Number of ID cases:*  In 2002  Hispanic 23 of 49 ASD cases  White 64 of 178 ASD cases  In 2004  Hispanic 19 of 34 ASD cases  White 21 of 78 ASD cases  In 2006  Hispanic 61 of 127 ASD cases  White 95 of 279 ASD cases  *ID present: absent ratio in children with ASD:*  In 2002  White 0.56:1  Hispanic 0.88:1  In 2004 (p<0.005)  Whit 0.37:1  Hispanic **1.27:1**  In 2006 (p<0.05)  White 0.52:1  Hispanic **0.92:1** | | | - |
| Haglund (2011) | Malmoe,  Sweden | Nested case-control  (Clinical record,  Linked to the birth register,  Children born 1980-2005 (N = 68 964),  157 cases,  Controls matched by  birth place and year of birth)  NOS: 7 | Autistic disorder DSM-IV or childhood autism ICD-10.  (92/138 (67%)  had mental  retardation) | Migration  (descendants of migrants):  Maternal  country of birth | *Number of cases/ total-number:*  Maternal country of birth and paternal  nationality:  Mother born in Sweden  Father Swedish 69/ 45,252  Father not Swedish 3/ 2,616  Mother not born in Sweden  Father Swedish 49/ 12,879  Father not Swedish 36/ 8,217  Maternal country of birth:  Sweden 72/ 47,868  Other Nordic countries 2/ 1,764  Western Europe/USA 6/ 1,332  Previous Eastern Europe 24/ 7,199  Sub-Saharan Africa 11/ 1,019  Middle East/North Africa 24/ 6,064  East Asia 8/ 1,574  South or Central America 6/ 1,229  Unknown/Other 4/ 915  *OR (95% CI):*  Maternal country of birth and paternal  nationality:  Mother born in Sweden  Father Swedish 1.0  Father not Swedish 0.8 (0.2-2.4)  Mother not born in Sweden  Father Swedish **2.5 (1.7-3.6)**  Father not Swedish **2.9 (1.9-4.3)**  Maternal country of birth:  Sweden 1.0  Other Nordic countries 0.8 (0.2-3.1)  Western Europe/USA **3.0 (1.3-6.9)**  Previous Eastern Europe **2.2 (1.4-3.5)**  Sub-Saharan Africa **7.3 (3.8-13.7)**  Middle East/North Africa **2.6 (1.7-4.2)**  East Asia **3.4 (1.6-7.1)**  South or Central America **3.3 (1.4-7.5)**  Unknown/Other **2.9 (1.1-8.0)**  Mother born outside Sweden **2.7 (2.0-3.7)** | *Adjusted OR (95% CI):*  Maternal country of birth and paternal nationality:  Mother born in Sweden  Father Swedish 1.0  Father not Swedish 0.7 (0.2-2.2)  Mother not born in Sweden  Father Swedish **2.1 (1.4-3.1)**  Father not Swedish **2.6 (1.7-3.9)**  Maternal country of birth:  Sweden 1.0  Other Nordic countries 0.8 (0.2-3.3)  Western Europe/USA **2.8 (1.2-6.5)**  Previous Eastern Europe **2.1 (1.3-3.3)**  Sub-Saharan Africa **5.6 (2.9-10.6)**  Middle East/North Africa **2.0 (1.2-3.2)**  East Asia **2.9 (1.4-6.1)**  South or Central America **3.1 (1.3-7.1)**  Unknown/Other **3.0 (1.1-8.3)**  *Additionally adjusted OR (95% CI):*  Mother born outside Sweden **2.2 (1.6-3.0)** | | Maternal age ≥40 years, birth <37 weeks, and  gestational age-adjusted weight  Standard Deviation (SD) scores (continuous)  Additionally adjusted for gender, parity, and maternal smoking |
| Barnevik-Olsson  (2010) | Stockholm,  Sweden | Cross-sectional  (Habilitation centers,  Compared with general population,  250 cases born 1999–2003)  NOS: 4 | Autism or PDD-NOS  (all had learning  disabilities) | Migration (Both migrants and descendants of migrants):  Children´s origin – Somali (born in Somalia or  Sweden, with both parents born abroad and at least one parent coming from Somali) or non-Somali | *Number of cases/ total-number:*  Somalian 18/ 1,836  Non-Somali 232/ 111,555  *Prevalence:* p<0.001  Somalian **0.98%**  Non-Somali 0.21% | | | - |
| Barnevik-Olsson  (2008) | Stockholm,  Sweden | Cross-sectional  (Habilitation centers,  Compared with general population,  501 cases born 1988-1998)  NOS: 4 | Autism or PDD-NOS  (all had learning  disabilities) | Migration (Both migrants and descendants of migrants):  Children´s origin – Somali (born in Somalia or  Sweden, with both parents born abroad and at least one parent coming from Somali) or non-Somali | *Number of cases/ total-number:*  Somalian 17/ 2,437  Non-Somali 484/ 250,565  *Prevalence (95% CI):* p<0.001  Somalian **0.7% (0.3-1.03)**  Non-Somali 0.19% (0.18-0.21) | | | - |
| Williams  (2008) | New South  Wales,  Australia | Case-control  (Surveillance,  Children born 1990-1999,  182 cases)  NOS: 5 | Autistic disorder DSM-IV | Migration  (descendants of migrants):  Maternal  country of birth | *Number of cases/ controls:*  Mother born outside Australia 65/ 23,121  Mother born in Australia 117 / 62,564  *OR (95% CI):*  Mother born outside Australia **1.5 (1.1-2.1)**  Mother born in Australia 1.0 | *Adjusted OR (95% CI):*  Mother born outside Australia 1.4 (1.0-1.9)  Mother born in Australia 1.0 | | Sex, premature,  mother > 35 years |
| Lauritsen  (2005) | Denmark | Cohort  (Register,  Children born 1984-1998 (n=943 664),  818 cases diagnosed by 2002)  NOS: 9 | Childhood autism (ICD-10: F84.0) or Atypical autism  (ICD-10: F84.1) | Migration  (descendants of migrants):  Maternal  country of birth  - Additional  analyses:  maternal and  paternal region of birth | *Number of cases:*  Maternal country of birth:  Denmark 688  Other Scandinavia and Europe 50  Outside Europe 78  Parental countries of births:  Mother and father not born in the same country  109  Mother and father born in the same country  690  *Incidence (per 10,000 person years at risk):*  Maternal country of birth:  Denmark 1.72  Other Scandinavia and Europe 2.36  Outside Europe 3.51  Parental countries of births:  Mother and father not born in the same country  3.00  Mother and father born in the same country  1.72 | *Adjusted RR (95% CI):*  Maternal country of birth: p = 0.03  Denmark 1.00  Other Scandinavia and Europe 1.02 (0.75-1.39)  Outside Europe **1.42 (1.10-1.83)**  Parental countries of births: p = 0.01  Mother and father not born in the same country  **1.36 (1.08- 1.71)**  Mother and father born in the same country  1.00  Additional analyses:  Both parents born in Denmark 1.00  Both parents born abroad 1.17 (0.90–1.51)  Only father born abroad 1.15 (0.84–1.58)  Only mother born abroad **1.77 (1.22–2.34)** | | Age and its  interaction with  gender, calendar  year of diagnosis, maternal and  paternal age, maternal, paternal and sibling psychiatric disorder, fathers  identity known,  urbanization and if mother and father were born in the same country |
| Hultman  (2002) | Sweden | Nested case-control  (Register,  Children born 1974-1993,  408 cases with the diagnosis at age <10 in 1987-1994,  2040 controls matched on sex, year and hospital of  birth)  NOS: 8 | Infantile autism  ICD-9. PDD-NOS not included | Migration  (descendants of migrants):  Mother’s  country of birth | *Number of cases/ controls:*  Nordic 351/ 1,890  Europe and North America 17/ 58  Outside Europe and North America 35/ 72  *OR (95% CI):*  Nordic 1.0  Europe and North America 1.6 (0.9-2.9)  Outside Europe and North America **2.9 (1.9-4.5)** | *Adjusted OR (95% CI):*  Nordic 1.0  Europe and North America 1.1 (0.5-2.5)  Outside Europe and North America **3.0 (1.7-5.2)** | | Maternal age,  parity, smoking  during pregnancy,  hypertension,  diabetes, bleeding in pregnancy, mode  of delivery, season of birth, gestational age, birth weight, Apgar score at 5 minutes, congenital malformations |
| Morton (2002) | Southern Derbyshire, UK | Cross-sectional  (Child development center,  Population estimated using a census,  Children aged <20 (n=116 312),  18 cases)  NOS: 4 | ASD except milder forms such as Asperger’s syndrome | Ethnic minority status | *Number of cases/ total-number:*  Mixed group (including European (95%), Black Caribbeans, Black Africans, Chinese and Bengalis)  9/ 110,075  Indian 2/ 3,513  Pakistani 7/ 2,724  *Estimated prevalence (95% CI):*  Mixed group^*^ 0.72 per 1000 (0.39-1.30)  Indian 0.57 per 1000 (0.16-2.07)  Pakistani 2.57 per 1000 (1.25-5.30) | | | - |
| Gillberg  (1996) | Goteborg  and Bohuslan,  Sweden | Cross-sectional  (Record linkage,  Children born 1975-1984 (n=78106),  55 cases)  NOS: 4 | Autistic disorder  DSM-III | Migration (Both migrants and descendants of migrants):  Migration status of at least one parent | 15/55 (27%) cases had one parent born abroad as compared to 26.2% in general population (not significant)  **11/15 (20%)** cases had one parent from outside Northern Europe as compared to 3.2% in general population (p<0.001) | | | - |
| Gillberg  (1987) | Goteborg  And  Bohuslan,  Sweden | Cross-sectional  (?,  Compared with general population,  35 cases with age < 10)  NOS: 4 | Infantile autism DSM-III | Migration (Both migrants and descendants of migrants):  Parental migration status | 30% (6/20) cases in Goteborg had one parent born abroad as compared to 25.7% in general population (not significant)  **0% (0/15)** children from the rural county had immigrant parents as compared to 25.7% in general population (p<0.001) | | | - |
| ASD = autism spectrum disorder; ID = intellectual disability; NOS = Newcastle-Ottawa Scale; PDD-NOS = pervasive developmental disorder-not otherwise specified; DSM = Diagnostic and Statistical Manual of Mental Disorders; ICD = International Classification of Diseases; RR = risk ratio; OR= odds ratio; CI = confidence interval | | | | | | | | |

| **Table S7. Any ID** | | | | | | | |
| --- | --- | --- | --- | --- | --- | --- | --- |
| **First**  **author**  (publica-tion data) | **Location** | **Design**  (Source of data, Population,  Sample size and description)  **Study quality with NOS** | **Case ascertainment** | **Exposure** (migration: either migrants or descendants of migrants) | **Results** | | **Adjustment factor** |
| Kim Van Naarden (2015) | Atlanta, USA | Cross-sectional  (Multiple-source surveillance (MADDSP),  Population estimated using a census,  Children aged 3-10 in 1991-1994 and children aged 8 in 1996-2010,  5590 cases)  NOS: 4 | Intellectual disability  defined as IQ<70 | Ethnic minority status | *Proportions of cases (1991-2010):*  White non-Hispanic 27.7% of 5,590 ID cases  Black non-Hispanic 59.2% of 5,590 ID cases  Hispanic 8.0% of 5,590 ID cases  Asian non-Hispanic 3.1% of 5,590 ID cases  *Prevalence (1991-2010):*  White non-Hispanic 8.4 per 1000  Black non-Hispanic 17.9 per 1000  Hispanic 11.7 per 1000  Asian non-Hispanic 8.9 per 1000 | | - |
| Emerson (2012) | UK | Cross-sectional  (School census,  Children aged 7-15 in 2008 (n=5 180 550),  Case 1.5% of the total sample)  NOS: 4 | Moderate, severe, and profound multiple learning difficulties according to a formal statement of special educational needs | Ethnic minority status | *Number of cases/ total-number:*  African -/ 2.5% of the total sample  Other Asian Background -/ 1.1% of the total sample  Other Black background -/ 0.5% of the total sample  Other ethnic group -/ 1.1% of the total sample  Other Mixed Background -/ 1.2% of the total sample  Other White background -/ 3.1% of the total sample  Bangladeshi -/ 1.3% of the total sample  Caribbean -/ 1.4% of the total sample  Chinese -/ 0.4% of the total sample  Gypsy/Romany -/ 0.1% of the total sample  Indian -/ 2.3% of the total sample  Irish -/ 0.4% of the total sample  Pakistani -/ 3.1% of the total sample  Traveller of Irish heritage -/ 0.1% of the total sample  White and Asian -/ 0.7% of the total sample  White and Black African -/ 0.3% of the total sample  White and Black Caribbean -/ 1.2% of the total sample  White British -/ 78.2% of the total sample  *OR (95% CI):* p<0.001  Moderate LD:  African **0.39 (0.36-0.42)**  Other Asian Background **0.38 (0.33-0.42)**  Other Black background **0.65 (0.57-0.73)**  Other ethnic group **0.38 (0.34-0.42)**  Other Mixed Background **0.66 (0.61-0.73)**  Other White background **0.54 (0.51-0.58)**  Bangladeshi **0.47 (0.43-0.53)**  Caribbean **0.74 (0.69-0.79)**  Chinese **0.36 (0.29-0.46)**  Gypsy/Romany **1.94 (1.68-2.24)**  Indian **0.55 (0.51-0.59)**  Irish **0.78 (0.67-0.91)**  Pakistani **0.76 (0.72-0.80)**  Traveller of Irish heritage **1.91 (1.55-2.34)**  White and Asian **0.60 (0.53-0.68)**  White and Black African **0.56 (0.48-0.67)**  White and Black Caribbean **0.72 (0.66-0.78)**  White British 1.00  Severe LD:  African 1.05 (0.96-1.14)  Other Asian Background 1.08 (0.95-1.24)  Other Black background 1.08 (0.90-1.30)  Other ethnic group 0.88 (0.77-1.01)  Other Mixed Background 1.02 (0.90-1.16)  Other White background **0.80 (0.72-0.88)**  Bangladeshi 1.15 (1.02-1.30)  Caribbean 1.02 (0.90-1.14)  Chinese 1.05 (0.83-1.34)  Gypsy/Romany 1.22 (0.90-1.64)  Indian 1.02 (0.92-1.13)  Irish 0.82 (0.63-1.06)  Pakistani **1.34 (1.24-1.44)**  Traveller of Irish heritage 1.40 (0.94-2.10)  White and Asian 0.89 (0.75-1.07)  White and Black African 0.77 (0.60-1.00)  White and Black Caribbean **0.69 (0.59-0.79)**  White British 1.00  Profound multiple LD:  African 1.03 (0.87-1.23)  Other Asian Background 1.20 (0.94-1.53)  Other Black background 1.32 (0.95-1.84)  Other ethnic group 1.18 (0.94-1.49)  Other Mixed Background 1.09 (0.86-1.38)  Other White background 1.05 (0.90-1.22)  Bangladeshi **1.46 (1.18-1.80)**  Caribbean 0.91 (0.71-1.16)  Chinese 1.01 (0.63-1.60)  Gypsy/Romany 1.21 (0.65-2.25)  Indian 1.14 (0.95-1.36)  Irish 1.15 (0.75-1.74)  Pakistani **2.38 (2.12-2.67)**  Traveller of Irish heritage 1.50 (0.67-3.36)  White and Asian 0.98 (0.71-1.36)  White and Black African 0.95 (0.60-1.49)  White and Black Caribbean 0.71 (0.53-0.94)  White British 1.00 | | - |
| McGrother (2002) | Leicesters-hire,  UK | Cross-sectional  (Register based on interviews,  Population from the census,  2256 South Asian and white cases)  NOS: 4 | Intellectual disability defined by "Heber 1959" and WHO 1992 | Ethnic minority status  (74.1 % of South Asian were migrants) | *Prevalence:*  South Asian 3.20 per 1000  White 3.62 per 1000  *Prevalence ratio for South Asians compared with whites (95% CI):*  Age 20-29 1.04 (0.81-1.32)  Age 30-39 **0.65 (0.49-0.87)**  Age 40-49 0.82 (0.56-1.20)  Age 50-59 **0.19 (0.07-0.52)**  All ages 0.88 (0.75-1.04) | | - |
| Morton (2002) | Southern Derbyshire, UK | Cross-sectional  (Child development center, Population estimated using a census,  Children aged <20 (n=116 312),  76 cases)  NOS: 4 | Severe learning disability defined as IQ<50 | Ethnic minority status | *Number of cases/ total-number:*  Mixed group (including European (95%), Black Caribbeans, Black Africans, Chinese and Bengalis) 42/ 110,075  Indian 7/ 3,513  Pakistani 27/ 2,724  *Estimated prevalence (95% CI):*  Mixed group^*^ 3.38 per 1000 (2.63-4.17)  Indian 1.99 per 1000 (0.97-4.11)  Pakistani **9.91 per 1000 (6.82-14.38)** | | - |
| Croen  (2001) | California,  USA | Retrospective cohort  (Regional centers,  Linked to the birth register,  Children born 1987-1994  (n= 4 590 333),  11 114 cases)  NOS: 9 | Idiopathic mental  retardation without  autism or cerebral palsy.  MR defined by the  California Department of  Developmental Services.  Mild (IQ 50-70), severe (IQ < 50), or unspecified  according to ICD-9 | Migration  (descendants of migrants):  Maternal birthplace  Ethnic minority status:  Maternal  race/ethnicity | *Number of cases/ total-number:*  Migration:  California 5,077/ 1,812,955  Other United States 2,042/ 883,320  Mexico 2,324/ 1,088,144  Other 1,259/ 591,470  Ethnic minority status:  White 4,189/ 1,884,556  Hispanic 4,239/ 1,845,765  Black 1,650/ 367,611  Asian 518/ 267,685  *Rate ratio (95% CI):*  Migration:  -Mild MR:  California 1.0  Other United States **0.8 (0.7-0.8)**  Mexico **0.7 (0.6-0.7)**  Other **0.6 (0.6-0.7)**  -Severe MR:  California 1.0  Other United States 1.0 (0.8-1.1)  Mexico 1.2 (1.1-1.3)  Other 1.1 (0.9-1.2)  Ethnic minority status:  -Mild MR:  White 1.0  Hispanic 1.0 (0.9-1.0)  Black **2.1 (1.9-2.3)**  Asian **0.6 (0.5-0.7)**  -Severe MR:  White 1.0  Hispanic **1.4 (1.3-1.6)**  Black **2.0 (1.7-2.4)**  Asian **1.4 (1.1-1.8)** | *Adjusted rate ratio (95% CI):*  Migration:  -Mild MR:  California 1.0  Other United States **0.8 (0.7-0.8)**  Mexico **0.6 (0.5-0.6)**  Other **0.7 (0.6-0.8)**  -Severe MR:  California 1.0  Other United States 1.0 (0.8-1.1)  Mexico 0.9 (0.8-1.1)  Other 0.9 (0.8-1.1)  Ethnic minority status:  -Mild MR  White 1.0  Hispanic 0.9 (0.8-1.0)  Black **1.5 (1.4-1.7)**  Asian **0.7 (0.6-0.8)**  -Severe MR:  White 1.0  Hispanic 1.2 (1.0-1.4)  Black **1.6 (1.4-1.9)**  Asian 1.3 (1.0-1.7) | Child sex, Birth  weight, Plurality,  Parity, Maternal age,  Maternal education,  Maternal race/ethnicity  Child sex, Birth  weight, Plurality,  Parity, Maternal age,  Maternal education,  Maternal birth place |
| Fernell (1998) | Botkyrka, Sweden | Cross-sectional  (Paediatric clinic,  Population from census 1995, Children born 1979-1992 (n= 14 138),  64 cases aged 0-4)  NOS: 3 | Severe mental retardation DSM-IV (IQ < 50-55) | Migration (both migrants and descendants of migrants): parental nationality | *Number of cases/ total-number:*  European (70% both Swedish parents) 33/ 8,893  Non-European* 31*/ 5,245  * Turkey 12; Iraq 2; Pakistan 4; Lebanon 4; Africa 1; South America 4; Syria 1; Malaysia 1.  *Prevalence for severe MR (95% CI):*  European (70% both Swedish parents) 3.7 per 1000 (2.1-5.3)  Non-European 5.9 per 1000 (4.3-7.5) | | - |
| Emerson (1997) | Metropolitan  Boroughs, UK | Cross-sectional  (Multiple source,  Population from the census,  221 Asian cases)  NOS: 3 | Learning disabilities according to health or educational services/ project staff/ a pre-existing Case Register | Ethnic minority status | *Number of cases/ total-number:*  Asian 148/ 20,990 in project 1 and  73/ 11,377 in project 2  *Prevalence for mild or moderate / severe learning disability:*  Age 0-4  Asian 0.00 per 1000 / 1.33 per 1000  Non-Asian 2.98 per 1000 / 1.01 per 1000  Age 5-14  Asian 2.65 per 1000 / 8.83 per 1000  Non-Asian 1.89 per 1000 / 3.22 per 1000  Age 15-24  Asian 3.30 per 1000 / 4.94 per 1000  Non-Asian 4.84 per 1000 / 1.64 per 1000  Age 25-34  Asian 3.22 per 1000 / 5.81 per 1000  Non-Asian 5.27 per 1000 / 1.95 per 1000 | | - |
| Drews  (1995) | Atlanta,  USA | Case-control  (Multiple-source surveillance (MADDS),  Children aged 10,  316 cases,  563 controls randomly selected from school records)  NOS: 6 | Isolated mental  retardation (without other neurological conditions): mild IQ 50-70 and severe IQ <50 | Ethnic minority status | *Number of cases/ controls:*  Mild MR:  White 98/ 296  Black 232/ 267  Severe MR:  White 50/ 296  Black 78/ 267  Any MR:  White 80/ 296  Black 236/ 267 | *Adjusted OR (95% CI):*  Mild MR:  White 1.0  Black **2.1 (1.4-3.1)**  Severe MR:  White 1.0  Black **3.8 (1.5-9.5)**  Any MR:  White 1.0  Black **2.3 (1.6-3.3)** | Sex, maternal age,  birth order, economic status, maternal education |
| ID = intellectual disability; NOS = Newcastle-Ottawa Scale; LD = learning difficulties; MR = mental retardation; OR= odds ratio; CI = confidence interval | | | | | | | |
